# The skeletome of the red coral *Corallium rubrum* indicates an independent evolution of biomineralization process in octocorals

**DOI:** 10.1186/s12862-020-01734-0

**Published:** 2021-01-11

**Authors:** Nathalie Le Roy, Philippe Ganot, Manuel Aranda, Denis Allemand, Sylvie Tambutté

**Affiliations:** 1 Centre Scientifique de Monaco, 8 Quai Antoine 1er, Monaco, MC 98000 Monaco; 2 Red Sea Research Center, King Abdullah University of Science and Technology (KAUST), Thuwal, Saudi Arabia; 3 Present Address: BOA UMR83, INRAe Centre Val de Loire, 37380 Nouzilly, France

**Keywords:** *Corallium rubrum*, Biomineralization, Axial skeleton, Sclerites, Organic matrix, Proteomics, Evolution

## Abstract

**Background:**

The process of calcium carbonate biomineralization has arisen multiple times during metazoan evolution. In the phylum Cnidaria, biomineralization has mostly been studied in the subclass Hexacorallia (i.e. stony corals) in comparison to the subclass Octocorallia (i.e. red corals); the two diverged approximately 600 million years ago. The precious Mediterranean red coral, *Corallium rubrum*, is an octocorallian species, which produces two distinct high-magnesium calcite biominerals, the axial skeleton and the sclerites. In order to gain insight into the red coral biomineralization process and cnidarian biomineralization evolution, we studied the protein repertoire forming the organic matrix (OM) of its two biominerals.

**Results:**

We combined High-Resolution Mass Spectrometry and transcriptome analysis to study the OM composition of the axial skeleton and the sclerites. We identified a total of 102 OM proteins, 52 are found in the two red coral biominerals with scleritin being the most abundant protein in each fraction. Contrary to reef building corals, the red coral organic matrix possesses a large number of collagen-like proteins. Agrin-like glycoproteins and proteins with sugar-binding domains are also predominant. Twenty-seven and 23 proteins were uniquely assigned to the axial skeleton and the sclerites, respectively. The inferred regulatory function of these OM proteins suggests that the difference between the two biominerals is due to the modeling of the matrix network, rather than the presence of specific structural components. At least one OM component could have been horizontally transferred from prokaryotes early during Octocorallia evolution.

**Conclusion:**

Our results suggest that calcification of the red coral axial skeleton likely represents a secondary calcification of an ancestral gorgonian horny axis. In addition, the comparison with stony coral skeletomes highlighted the low proportion of similar proteins between the biomineral OMs of hexacorallian and octocorallian corals, suggesting an independent acquisition of calcification in anthozoans.

## Background

**Fig. 1:**
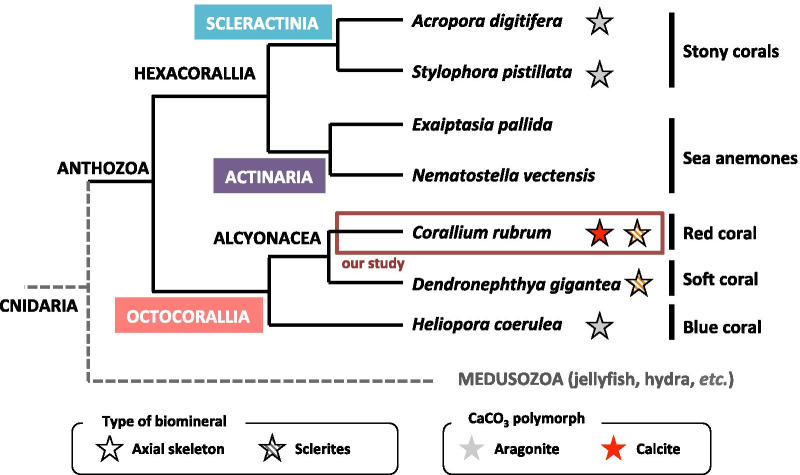
Phylogenetic relationship of anthozoan species used in the present study. Anthozoans are divided into two subclasses: Octocorallia and Hexacorallia. In Octocorallia, 3 species are represented: the red coral *Corallium rubrum* (order Alcyonacea), which is the model of the present study (red frame), and 2 other species, the soft coral *Dendronephthya gigantea* (order Alcyonacea) and the blue coral *Heliopora coerulea* (order Helioporacea). In Hexacorallia, 4 species are represented: 2 stony corals (order Scleractinia) and 2 sea anemones (order Actinaria). Phylogeny is based on www.tolweb.fr. The presence of axial skeleton and/or sclerites and the CaCO_3_ polymorph are indicated by colored stars

Biomineralization is a widespread process in metazoans, and biomineral structures provide a multitude of functions (protection, maintenance, etc.) [1]. Among marine calcifiers, corals represent one major group. They produce a CaCO_3_ exoskeleton to sustain the vertical and horizontal growth of the coral colony. The vernacular term “coral” refers to calcifying organisms within the phylum Cnidaria. Anthozoa, a class of Cnidaria, encompasses two major subclasses (Fig. 1): Hexacorallia and Octocorallia. Among hexacorallians only stony corals (order Scleractinia) are calcifiers, they are the basis of the edification of coral reefs [2]. On the other hand, octocorallians, with very few exceptions, are all calcifiers in the sense that they produce sclerites, i.e. micrometric calcified skeletal elements found throughout the tissues [3]. Many octocorallians, such as gorgonians (order Alcyonacea), also have a central horny axis composed of sclerotized collagen [4, 5]. In red corals (order Alcyonacea), the central axis is a calcified axis [6, 7]. Hexacorallians and octocorallians diverged approximately 600 Mya [8, 9]. Comparison of the mechanisms that govern the production of calcium carbonate (CaCO_3_) biominerals in hexacorallians (scleractinians) and octocorallians is thus expected to shed light on the conservation of the protein toolkit used by these organisms for making their biocalcification. Moreover, studying corals that can produce distinct skeletal structures holds the potential to provide novel insight into the evolutionary aspect of biomineralization.

In this respect, the red coral *Corallium rubrum* is a model of interest for the study of biomineralization. This species, endemic to the Mediterranean Sea, is noteworthy for its intense red color but also because it produces two distinct biominerals, the axial skeleton and the sclerites. The sclerites are formed by specialized cells named scleroblasts and are spread within the mesoglea [10, 11]. Axial skeleton formation is the result of two separated processes that produce two distinct structures, the medullar and the annular regions. First, the axial skeleton extends at the apex of the branches by an aggregation of the sclerites (longitudinal branch extension), forming the medullar part [7, 12–14]. Then, calcite is deposited concentrically around the medulla by the axial skeleton epithelium. While the axial skeleton has a clear role in supporting the coral colony, the sclerites have suggested roles in protection against predators, waste storage, and as initiation sites for axial skeleton formation [14]. Both biominerals are composed of a high-magnesium calcite polymorph as opposed to the aragonite skeletons of stony corals [7, 15–18]. In *C. rubrum*, the organic matrix (OM; 1–2% of the biomineral), which can be dissociated as a water-soluble organic matrix (SOM) and water-insoluble organic matrix (IOM), essentially contains proteins, glycoproteins and polysaccharides [19]. The term “organic matrix” refers here to the components of the matrix forming the scaffold involved in the process of biomineralization, i.e. the structural components displaying extracellular matrix (ECM) properties (scaffold), as well as the components required for the precipitation of CaCO_3_ [20, 21]. In cnidarians, the process of OM assembly remains to be elucidated.

Our understanding of the role of OM components in biomineralization is derived from many in vitro and a few in vivo experiments. These molecules can stabilize amorphous calcium carbonate and control the nucleation, the orientation and the polymorph selection of the crystal [20, 22–25]. So far organic matrices of *C. rubrum* have been electrophoretically characterized and hitherto only one OM protein, scleritin, has been sequenced [13, 19, 26–28]. Recently, the first investigation of the OM proteome of the two biominerals was performed in the octocorallian species *Corallium konojoi* (Japanese red and pink coral species). The authors showed a low similarity of proteins between the SOM of the axial skeleton and the sclerites, with only 9 out of the 147 identified proteins being common to both biominerals. However, at the time of this study, neither octocorallian transcriptomes nor genomes were available, and the authors had to rely on phylogenetically distant hexacorallian transcriptomes to identify their octocorallian OM proteins [29]. Hence, identification of the proteome components may have been artefactual in parts.

In the present study, we performed high-resolution mass spectrometry analysis to characterize the proteomes of the SOM and the IOM of the axial skeleton, and the sclerites of *C. rubrum*. By analyzing these results in combination with the *C. rubrum* transcriptome, we have identified a total of 102 OM proteins that were further manually curated and categorized according to their possible function. Also, we analyzed their conservation with other sequenced anthozoans: (i) 3 octocorals, (ii) 4 scleractinian species known to have a fast skeletal growth rate, and (iii) 3 non-calcifying sea anemones.

## Results

### The proteome of *Corallium rubrum* biominerals

For each biomineral, the extraction process produced 2 OM fractions, the water-soluble organic matrix (SOM) and the water-insoluble organic matrix (IOM). After 1D SDS-PAGE electrophoresis and trypsin digestion of the SOM and IOM of the two biominerals, spectra from tandem mass spectrometry were analyzed and interrogated against our in-house transcriptome assembly (CrubmRNA.fasta; data available at http://data.centrescientifique.mc/CSMdata-crubrum_data.html) using Mascot. We identified and retained for further analysis 107 proteins with a minimum of 2 unique peptides per protein sequence in at least one of the 4 fractions.

The 107 protein sequences were then annotated using the Blast2GO pipeline as well as other prediction tools (see Methods). They were named CR_n, in the descending order of their unique peptide count. According to our BLAST analysis, out of the 107 proteins, 34 were distributed into 9 groups of similar proteins (Additional file 1). Two groups were particularly represented within the red coral skeletome; the collagen-like proteins (10 proteins), and the agrin-like proteins (8 proteins). Both families are known as extracellular matrix (ECM) proteins [30, 31]. Also of interest, 20 proteins did not match with known proteins (no BLAST hit). Finally, when analyzed by a BLAST search against NCBI Metazoa and Prokaryota RefSeq databases (April 2020), one protein (CR_22) exhibited high similarity with prokaryote proteins, and with uncharacterized proteins from only one metazoan species: *Dendronephthya gigantea* (Octocorallia/Alcyonacea). Concurrent to the proteomic and transcriptomic project of the red coral, we are currently assembling the *C. rubrum* genome (PG, MA, DA, ST; personal communication). Although at a draft stage, we were able to map all the aforementioned 21 (20 + 1) proteins’ mRNA sequences to our *C. rubrum* genome assembly. Beside the fact that all proteins were encoded in the genome (Table 1), they also contained introns (except CR_91), suggesting that they were genuine *C. rubrum* proteins.

**Table 1:** *Corallium rubrum* proteins identified in the axial skeleton and the sclerites

CR_n	Transcript accession n°	Description	Protein family	Presence in the genome
CR_1	TRINITY_DN57230_c0_g1_i1	Scleritin (UOMP)		
CR_2	TRINITY_DN62338_c1_g1_i2	Sushi domain-containing 2-like	Mucin	
CR_3_4	scaff2294:c99220-62,422	CAP-like	CAP	Yes
CR_5	TRINITY_DN53149_c0_g1_i1	Collagen triple helix repeat	Collagen	Yes
CR_6	TRINITY_DN55882_c0_g1_i1	Fibrinogen-related		
CR_7	TRINITY_DN63002_c0_g1_i7	Low-density lipo receptor-related like 4	LRP	
CR_8	TRINITY_DN56567_c4_g1_i4	UOMP		Yes
CR_9	TRINITY_DN52968_c0_g1_i1	Collagen α -1(V) chain	Collagen	Yes
CR_10_28_60	scaff2907:c1055600-1,011,000	Agrin-1	Agrin	Yes
CR_11	TRINITY_DN58426_c1_g1_i1	?		
CR_12	TRINITY_DN58029_c4_g3_i1	Tyrosinase		
CR_13	TRINITY_DN58119_c0_g1_i1	?		
CR_14	TRINITY_DN62074_c1_g1_i7	Galaxin (UOMP)	Galaxin	
CR_15	TRINITY_DN63296_c1_g4_i3	Melanotransferrin-like		
CR_16	TRINITY_DN56632_c0_g3_i1	MMP12A	Peptidase (zinc)	
CR_17	TRINITY_DN52633_c0_g1_i1	Collagen α -1(XI) chain	Collagen	
CR_18	TRINITY_DN62446_c0_g1_i1	?		
CR_19	TRINITY_DN51464_c0_g1_i1	Agrin	Agrin	
CR_20	TRINITY_DN57322_c0_g1_i1	MMP12B	PEPTIDASE (zinc)	
CR_21	TRINITY_DN52133_c0_g1_i1	NA	Sugar-binding	Yes
CR_22	TRINITY_DN60196_c2_g6_i1	?	Sugar-binding	Yes
CR_23	TRINITY_DN61543_c0_g1_i1	Plexin domain-containing protein		
CR_24	TRINITY_DN64090_c0_g1_i6	Protocadherin like	Protocadherin	
CR_25	TRINITY_DN58830_c0_g1_i1	NA	Frizzled domain	Yes
CR_26	TRINITY_DN58830_c0_g4_i2	NA	Frizzled domain	Yes
CR_27	TRINITY_DN61967_c0_g2_i2	Galaxin (UOMP)	Galaxin	
CR_29	TRINITY_DN45133_c0_g1_i1	CruCA4		
CR_30	TRINITY_DN63986_c2_g1_i1	Peptidase S8	Peptidase (serine)	
CR_31	TRINITY_DN48716_c1_g1_i1	NA		Yes
CR_32	TRINITY_DN63619_c0_g1_i8	?		
CR_33	TRINITY_DN55669_c0_g3_i8	UOMP		Yes
CR_34	TRINITY_DN63069_c2_g1_i2	Protocadherin Fat 4	Protocadherin	
CR_35	TRINITY_DN58620_c3_g1_i1	Collagen α -1(III) chain	Collagen	Yes
CR_36	TRINITY_DN62341_c4_g3_i7	NA		Yes
CR_37	TRINITY_DN56000_c0_g2_i3	UOMP		Yes
CR_38	TRINITY_DN54322_c0_g1_i1	Collagen α -2(I) chain	Collagen	Yes
CR_39	TRINITY_DN53417_c0_g1_i4	Collagen α -1(VI) chain	Collagen	Yes
CR_40	TRINITY_DN62648_c2_g1_i1	Transmembrane protease serine 9	GliPR	
CR_41	TRINITY_DN41663_c0_g1_i1	Exostosin-3	GAG_elongation	
CR_42	TRINITY_DN63404_c0_g2_i1	Peptidyl-glycine α -amidating monooxygenase	PAL_PHM_PAM	
CR_43	TRINITY_DN48082_c0_g1_i1	Renin receptor		
CR_44	TRINITY_DN51483_c0_g1_i1	V-type proton ATPase subunit S1-like		
CR_45	TRINITY_DN63929_c2_g1_i5	Pentraxin	Sugar-binding	
CR_46	TRINITY_DN63365_c0_g1_i3	Peptidyl-α-Hydroxyglycine α-amidating lyase	PAL_PHM_PAM	
CR_47	TRINITY_DN62037_c1_g1_i1	NA	Sugar-binding	Yes
CR_48	TRINITY_DN63397_c3_g1_i3	UOMP		Yes
CR_49	TRINITY_DN61472_c0_g2_i3	Cytoplasmic Actin		
CR_50	TRINITY_DN60827_c2_g2_i3	Collagen α -2(V) chain	Collagen	
CR_51	TRINITY_DN63041_c1_g1_i2	Complement component C3 precursor		
CR_52	TRINITY_DN63149_c1_g3_i3	Cystatin	Peptidase inhibitor	
CR_53	TRINITY_DN58234_c0_g2_i1	Agrin	Agrin	
CR_54	TRINITY_DN61441_c0_g1_i3	Collagen α -1(II) chain isoform X2	Collagen	
CR_55	TRINITY_DN51187_c0_g2_i2	UOMP		
CR_56	TRINITY_DN63166_c7_g6_i1	Collagen α -1(I) chain-like	Collagen	
CR_57	TRINITY_DN54300_c1_g1_i1	Pentraxin	Sugar-binding	
CR_58	TRINITY_DN58073_c0_g1_i1	Pikachurin-like	Sugar-binding	
CR_59	TRINITY_DN64032_c0_g1_i3	α -2-Macroglobulin 1 isoform X2		
CR_62_76_82	scaff2512:c157700-123,800	Agrin-2	Agrin	Yes
CR_61	TRINITY_DN61195_c0_g1_i1	MMP13	Peptidase (zinc)	
CR_63	TRINITY_DN45588_c0_g1_i1	Tenascin-like		
CR_64	TRINITY_DN63439_c12_g3_i1	MMP12A	Peptidase (zinc)	
CR_65	TRINITY_DN56377_c0_g1_i2	CUB and sushi Domain-containing 3	Sugar-binding	
CR_66	TRINITY_DN51686_c0_g1_i1	Superoxide dismutase [Cu–Zn]		
CR_67	TRINITY_DN58794_c0_g8_i1	UBA52		
CR_68	TRINITY_DN63204_c1_g1_i3	Collagen α -1(I) chain	Collagen	
CR_69	TRINITY_DN63340_c1_g2_i1	Zinc transporter ZIP14		
CR_70	TRINITY_DN62875_c0_g2_i2	Protein kinase C-binding protein NELL1	Sugar-binding	
CR_71	TRINITY_DN43523_c0_g1_i1	?		
CR_72	TRINITY_DN53651_c2_g1_i1	UOMP		
CR_73	TRINITY_DN30803_c0_g2_i1	Calumenin	EFh_CREC_cab45	
CR_74	TRINITY_DN53562_c0_g1_i1	Bactericidal permeability-increasing-like		
CR_75	TRINITY_DN39424_c0_g1_i1	UOMP		Yes
CR_77	TRINITY_DN62341_c4_g3_i6	NA		Yes
CR_78	TRINITY_DN58182_c1_g1_i1	UOMP		
CR_79	TRINITY_DN61728_c0_g3_i8	Sialate O-acetylesterase		
CR_80	TRINITY_DN56386_c0_g1_i1	UOMP		
CR_81	TRINITY_DN61964_c1_g1_i4	Cathepsin Z	Peptidase (cysteine)	
CR_83	TRINITY_DN56964_c4_g1_i1	Thrombospondin-type laminin G domain and EAR repeat-containing protein		
CR_84	TRINITY_DN54518_c0_g1_i3	NA		Yes
CR_85	TRINITY_DN63555_c0_g1_i5	?	Sugar-binding	Yes
CR_86	TRINITY_DN43665_c0_g1_i1	45 kDa calcium-binding	EFh_CREC_cab45	
CR_87	TRINITY_DN63601_c0_g1_i2	Transforming growth factor-β-induced ig-h3-like		
CR_88	TRINITY_DN19382_c0_g1_i1	UOMP		Yes
CR_89	TRINITY_DN55648_c0_g1_i2	?		
CR_90	TRINITY_DN62899_c0_g1_i1	Peptidase S9	Peptidase (serine)	
CR_91	TRINITY_DN37336_c0_g1_i1	UOMP		Yes*
CR_92	TRINITY_DN60582_c0_g1_i2	Haem peroxidase	Haem peroxidase	
CR_93	TRINITY_DN30927_c0_g2_i1	Prolyl 4-hydroxylase-like	P4H	
CR_94	TRINITY_DN57626_c0_g3_i10	UOMP		Yes
CR_95	TRINITY_DN58599_c0_g1_i3	?		Yes
CR_96	TRINITY_DN12946_c0_g1_i1	Gamma-glutamyl-transpeptidase		
CR_97	TRINITY_DN55829_c0_g1_i3	NA	Sugar-binding	Yes
CR_98	TRINITY_DN57865_c0_g2_i3	von Willebrand factor, type A		
CR_99	TRINITY_DN64024_c5_g1_i3	Histone H4		
CR_100	TRINITY_DN59633_c0_g1_i1	Adhesion G-coupled receptor D1		
CR_101	TRINITY_DN55506_c0_g1_i1	Low-density lipo receptor-related 6	LRP	
CR_102	TRINITY_DN36837_c0_g1_i1	Histone H2B type 2-E-like		
CR_103	TRINITY_DN53166_c0_g1_i1	UOMP		Yes
CR_104	TRINITY_DN61990_c1_g1_i7	NA		Yes
CR_105	TRINITY_DN55043_c0_g1_i2	UOMP		
CR_106	TRINITY_DN59627_c2_g1_i13	UOMP		Yes
CR_107	TRINITY_DN61728_c0_g3_i1	Sialate O-acetylesterase		

Proteins are presented by: number of identified proteins (CR_n), accession number of the corresponding transcript from transcriptomic CrubmRNA.fasta database of the Centre Scientifique de Monaco, description of proteins, protein family and presence of encoding gene in *C. rubrum* genome. *UOMP* uncharacterized organic matrix protein; ?: unknown protein but containing characterized domains, *NA* not annotated; Yes*: presence in *C. rubrum* genome but no intron

To avoid redundancy, we manually cross-examined the sequences contained within each group/family against our *C. rubrum* genome draft assembly (PG, MA, DA, ST; personal communication) and another *C. rubrum* published transcriptome [32]. Several of the initially assembled transcripts turned out to be partial and were in fact portions of the same larger sequence. Indeed, CR_3 and CR_4 are 2 moieties of the same CAP sequence (cysteine-rich secretory proteins, antigen 5, and pathogenesis-related 1 protein) and the three CR_10, CR_28, CR_60 and the three CR_62, CR_76, CR_82 were portions of two large agrin-like proteins (Additional file 1, Additional file 2). The non-redundant protein set of the *C. rubrum* OM is thus 102 proteins (Table 1, Additional file 3). The rest of the analysis was performed on these 102 proteins.

### Sclerites vs axial skeleton organic matrix composition

The axial skeleton is formed by medullar part (occluding sclerites) and annular part (without sclerites). Relative contribution of medullar and annular parts in the axis is dependent on the colony age, since the medulla is of constant diameter whereas the annular part increases throughout time. Using 7 published axial skeleton cross-sections (Additional file 4) [7, 33], we estimated the proportion of the sclerites into the axial skeleton. The surface ratio between the medulla and the annula (easily discernible after staining, see references) showed that the medulla surface represented 5.37% (SEM ± 1.05%) of the annula (Additional file 4). In medulla, sclerites make up about half of the medullar area [14], which corresponds to a proportion of about 3% of the axis surface. Thus, in our study, the axial skeleton also contained proteins from the sclerite OM, although the overall proportion is low.

**Fig. 2:**
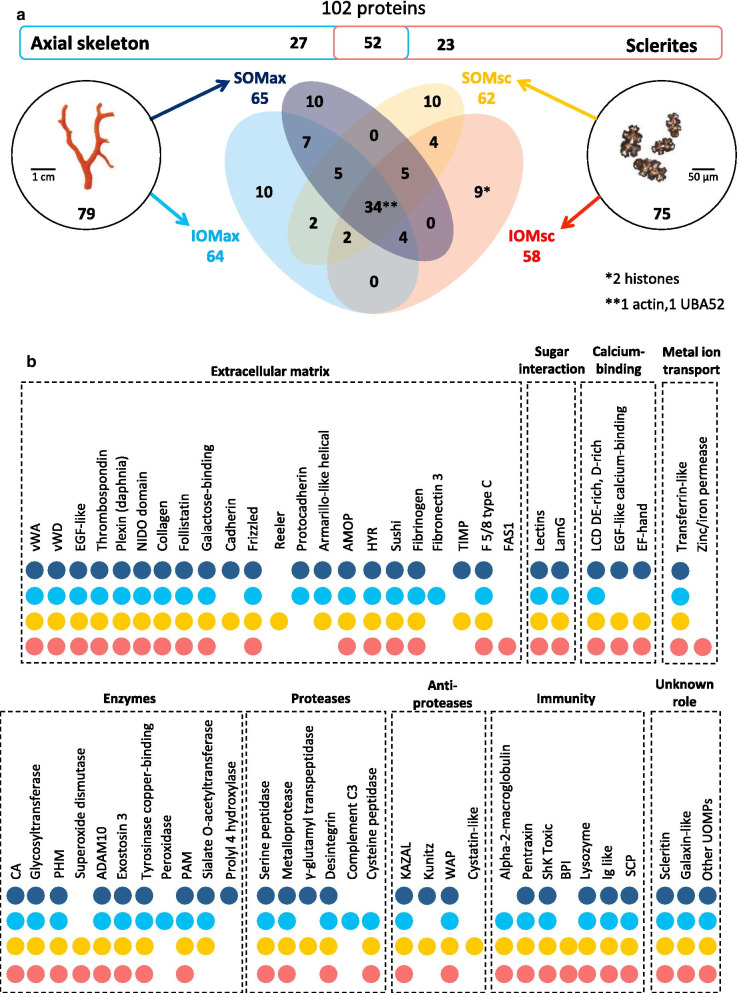
Distribution of the proteins identified in the organic matrix of the red coral axial skeleton and sclerites. **a** Among the total 102 proteins identified in the *C. rubrum* biominerals, 79 are present in the axial skeleton (left) and 75 are present in the sclerites (right), 52 being shared between the two. The Venn diagram shows the number of shared and non-shared proteins between the 4 extracted organic matrices: SOMax: soluble organic matrix of the axial skeleton, IOMax: insoluble matrix of the axial skeleton, SOMsc: soluble organic matrix of the sclerites, and IOMsc: insoluble organic matrix of the sclerites. **b** Protein domains identified in the proteome of the red coral biominerals and their distribution using circle representation in SOMax (dark blue), IOMax (blue), SOMsc (yellow) and IOMsc (red). *ADAM10* desintegrin and metalloproteinase, *AMOP* adhesion-associated domain present in MUC4 and other proteins, *BPI* bactericidal permeability-increasing protein/lipopolysaccharide-binding protein/cholesteryl ester transfer protein N-terminal domain, *CA* carbonic anhydrase, *EGF-like* epidermal growth factor, *EF-hand* a calcium-binding domain, *F5/8 type C* coagulation factor 5/8 C-terminal domain, *FAS1* fasciclin-like, *HYR* hyalin repeat, *IG-like* immunoglobulin-like, *KAZAL* serine protease inhibitor, *LamG* laminin G, *LCD* low complexity domain, *NIDO* extracellular domain of unknown function in nidogen, *PAM* peptidylglycine α-amidating monooxygenase, *PHM* peptidylglycine α-hydroxylating monooxygenase, *ShK toxic* stichodactyla toxin, *SCP* Cysteine-rich secretory proteins, antigen 5, and pathogenesis-related 1 proteins (CAP) superfamily proteins, *TIMP* tissue inhibitor of metalloproteinase, *vWA* von Willebrand type A, *vWD* von Willebrand type D, *UBA52* Ubiquitin A-52, *UOMPs* uncharacterized organic matrix proteins, *WAP* whey acidic protein ‘four-disulfide core’

We explored the distribution of OM proteins within the axial skeleton and the sclerites (Fig. 2a, Table 1). Among the 102 proteins composing the red coral proteome, the OM of the axial skeleton and the sclerites was composed of 79 and 75 proteins, respectively. Twenty-seven were specific to the axial skeleton whereas 23 were specific to the sclerites; 52 were common to both. These numbers correspond to proteins identified in individual fractions with a cut-off of minimum 2 peptides per protein. With a cut-off decreased to minimum of 1 peptide per protein, the 102 OM proteins would correspond to 92 and 84 proteins present in the axial skeleton and the sclerites, respectively, with 74 proteins common to both biominerals (Additional file 3 and Additional file 5). Nevertheless, we here favored the stringent cut-off (2 peptides/protein) in the rest of the analysis in order to avoid false positive protein identification in our samples [34].

The protein distribution between the SOM and IOM fractions of the organic matrix was somewhat equivalent in both the axial skeleton (65 SOM and 64 IOM proteins) and the sclerites (62 SOM and 58 IOM proteins, Fig. 2a). In the axial skeleton and the sclerites, only minorities of the proteins were specific to the IOM (10 and 9, respectively) or the SOM (10 and 10, respectively), and 34 were common to the SOM and IOM of both *C. rubrum* biominerals.

Using the prediction tools InterProScan and SMART, we analyzed the domain composition of proteins of the *C. rubrum* biominerals. We found domains such as vWA, vWD, EGF-like, thrombospondin, sushi, reeler, fibrinogen, fibronectin and protocadherin (Fig. 2b). We also noticed the presence of sugar-binding domains in 10 proteins (CR-21, 22, 45, 47, 57, 58, 65, 70, 85 and 97; Table 1). Interestingly, the fibronectin (CR_84), protocadherin (CR_24 and CR_34), and the peroxidase (CR_92) domains were found in proteins present in the axial skeleton but not in the sclerites. Finally, for 18 of the OM proteins, no domains were identified and they were classified as Uncharacterized Organic Matrix Proteins (UOMPs; Table 1).

We quantified the relative abundance of proteins (Additional file 3 sheet#1) in the four OM fractions using two label-free methods: exponentially modified protein abundance index (emPAI) and weighted spectral count (WS). According to emPAI values, the 4 proteins CR_1, CR_3-4, CR_5 and CR_10-28–60 were listed within the top 10 most abundant proteins in all 4 samples (i.e. SOM and IOM in both the axial skeleton and the sclerites; Additional file 6). We noticed that CR_1 (scleritin), previously identified in the OM of the sclerites of *C. rubrum* [13], was the highest abundant protein in SOM and IOM of the axial skeleton (emPAI: 32.011 and 30.225, respectively) and the sclerites (emPAI: 71.871 and 66.78, respectively), which is supported by WS results (Additional file 6). Although of unknown function and with no functional domain annotation, its high abundance in both biominerals emphases its importance in the biomineral formation of *C. rubrum*. CR_1 was followed distantly by CR_3-4 (CAP family protein, a prototype extracellular protein) that was more abundant in the axial skeleton OM than in the sclerites OM.

### Major protein families of the red coral OM

Among the 102 proteins found in the OM of *C. rubrum* biominerals, we choose to limit in this section those with potentially evolutionary interest.

#### Collagen-like proteins

**Fig. 3:**
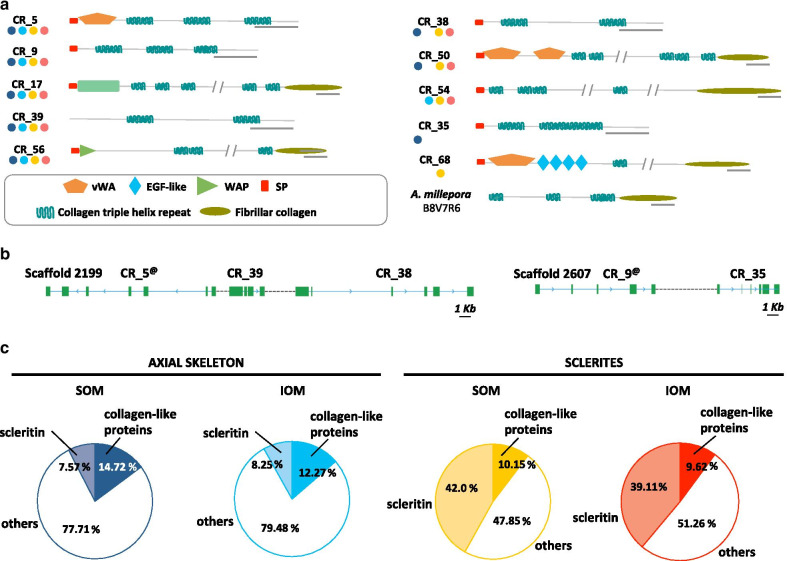
Schematic representation of the 10 collagen-like protein sequences identified in the proteome of the red coral biominerals. The collagen sequence identified in the *Acropora millepora* (UniProt accession number B8V7R6) organic matrix is included. **a** Schematic protein domain (described in the box below) representation of the different collagen proteins with their respective ID (CR_n). Colored circles indicate presence of the protein in different fractions as described in Fig. 2b. *EGF-like* epidermal growth factor-like, *SP* signal peptide, *TSPN* Thrombospondin N-terminal-like domain, *vWA* von Willebrand factor type A. Scale bars = 100 amino acids. **b** Genomic synteny of the CR_5, CR_39, and CR_38 genes and of the CR_9 and CR_35 genes; exons are in green boxes, introns are in blue lines, and intergenic sequences are in dotted lines. Scale bar = 1000 base pairs. **c** The overall proportion of collagen-like proteins and the most abundant protein, scleritin (CR_1), in each sample in percentage of weighted spectra

In the red coral, out of 10 collagen-like proteins, 8 were identified in the two biominerals (CR_5, 9, 17, 35, 38, 39, 50, 54, 56 and 68), one in the axial skeleton (CR_35) and one in the sclerites (CR_68) (Fig. 3a, Additional file 7). Collagens are ECM proteins that constitute a large family involved in the ultrastructural organization of the organic matrix network [35]. Most collagens contains a repeat motif of 3 amino acids (G–X–Y) and form fibrils after post-translation modification [36]. Initially, 12 proteins were similar to collagens according to the BLAST search results. However, 2 of these candidates, CR_6 and CR_98, were arguably assigned as collagens: they did not possess triplet repeats G–X–Y, but harbored fibrinogen-related and von Willebrand A domains, which are also present within collagen sequences of other species. As a consequence, they were automatically annotated as “collagen” (Additional file 8), but here, we did not consider these as such. The same holds true for two other proteins (P14 and P18) identified as collagen in the OM of the scleractinian coral *S. pistillata* [18]. However, with the completion of the *S. pistillata* genome [37], reassessment of these sequences shows that P14 is a coadhesin, and P18 a vWA domain-containing protein (Additional file 8). Finally, only one collagen-like sequence was identified in the biomineral of another scleractinian coral, *Acropora millepora*, which corresponds to a collagen containing G–X–Y motif (Acc. B8V7R6; [16]). In *C. rubrum*, the remaining 10 collagen-like proteins (CR_5, 9, 17, 35, 38, 39, 50, 54, 56 and 68) had typical collagen domains (Fig. 3a, Additional file 7). Five of them also had a C-terminal fibrillar collagen domain. The 5 without (CR_5, 38, 39 and CR_9, 35) were encoded on two gene clusters (Fig. 3b). Taken individually, none of these 10 collagen-like proteins accounted for the major protein of a biomineral fraction. However, when summed up (Fig. 3c), “collagen” was the most abundant protein of the axial skeleton OM (~ 14%). In the sclerites, collagen-like proteins were present at similar quantities (~ 10%), though scleritin was by far the most abundant OM protein (~ 40%). In vertebrates, collagens are classified in types according to their physico-chemical properties and tissue specific expression. Based on BLAST searches and domain signatures, the *C. rubrum* collagen-like proteins were α-type fibrillar assigned as type I, II, III, V, VI, and XI (Table 1). Of note, collagen type IV, which is characterized by its NC1 domain, is present in cnidarians [38] including *C. rubrum* (P. Ganot, personal communication), but was not detected in the biominerals’ OM.

#### Agrin-like proteins

**Fig. 4:**
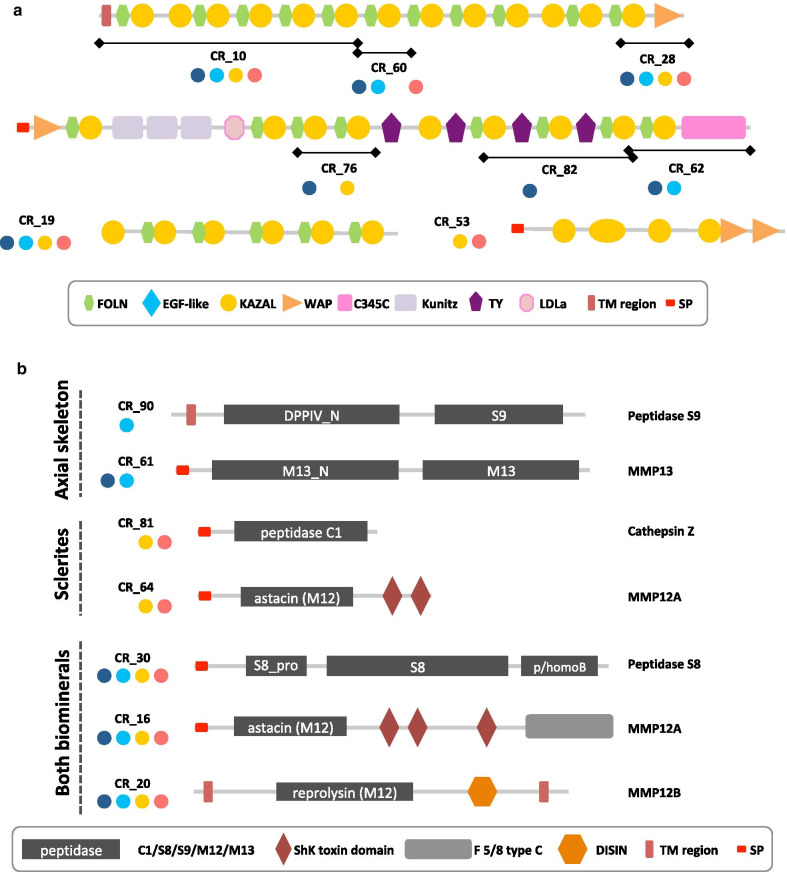
Schematic representation of the 7 proteases and the 4 agrin-like proteins identified in the proteome of the red coral biominerals. **a** The peptidase S9 (CR_90) and the matrix metallopeptidase 13 (MMP13, CR_61) are specific to the axial skeleton OM. The cathepsin Z (CR_81) and one MMP12A (CR_64) are specific to the sclerites. The peptidase S8 (CR_30), the other MMP12A (CR_16) and the MMP12B (CR_20) are found in both biominerals. **b** CR_10, CR_60 and CR_28 are part of the same agrin-1 protein. CR_76, CR_82 and CR_62 are part of the same agrin-2 protein. Proteome ID are indicated, circle representation in the different biominerals fractions is as in Fig. 2b, and protein domains are described in the box. *C345C* netrin C-terminal domain, *DISIN* disintegrin domain, *EGF-like* epidermal growth factor-like, *F5/8 type C* coagulation factor 5/8 C-terminal domain, *FOLN* follistatin-N-terminal domain-like, *LDLa* low-density lipoprotein receptor domain class A, *ShK toxin* stichodactyla toxin, *SP* signal peptide, *TM* transmembrane, *TY* thyroglobulin type I repeats, *WAP* four-disulfide core domain

The agrin-like protein family is the second most diversified family, in terms of number of genes, in the *C. rubrum* OM. In vertebrates, agrin is a single copy gene that produces different proteins through alternative splicing [39, 40]. Agrins are long proteoglycans (heavily glycosylated proteins) found in many extracellular environments. They are composed of two main parts, the N-terminal moiety, which is composed of a series of Foln/Kazal domains (Fig. 4a) where glycosaminoglycan (GAG) side chains are attached, and the C-terminal moiety, which contains binding sites for various ligands including LRP4 (Low-density Lipoprotein Receptor-related Protein 4), and dystroglycan, among others [39, 40]. Agrins are highly repetitive proteins, especially in their N-terminal moiety, and as such, their nucleotide sequence is difficult to assemble, especially with high-throughput RNAseq technologies using short reads. In our *C. rubrum* transcriptome assembly, we found different transcripts corresponding to pieces of agrin homologs (CR_10, 19, 28, 53, 60, 62, 76 and 82). However, when mapped to our draft genome and to the transcriptome of Pratlong [32], we found that CR_10, CR_60, and CR_28, and CR_76, CR_82 and CR_62 were parts of two agrin proteins, agrin-1 and agrin-2, respectively (Fig. 4a, Additional file 1 and Additional file 2). In all, we were able to identify 4 different agrin homologs in the OM of the *C. rubrum* biominerals. CR_10-28-60, CR_62-76-82 and CR_19 were present in both biominerals and CR_53 was only identified in the sclerite OM (Fig. 4a, Additional file 3 sheet#2). Although their protein sequence may yet be partial for some of them, all 4 corresponded to homologs of the N-terminal moiety of vertebrate agrins, i.e. the moiety carrying the sugar chains.

#### Matrix peptidases

We identified 7 peptidases in the *C. rubrum* OM: 4 matrix metallopeptidases (MMPs), 2 serine peptidases and 1 cysteine peptidase (Table 1, Fig. 4b). These proteases are not equally distributed between the 2 biominerals: the serine peptidases S9 (CR_90) and MMP13 (CR_61) were specific to the axial skeleton OM whereas the cysteine protease (CR_81) and one MMP12 (CR_64) were specific to the sclerite OM (the remaining 3 being present in both biominerals; Additional file 3 sheet#2).

#### Carbohydrate-binding proteins

**Fig. 5:**
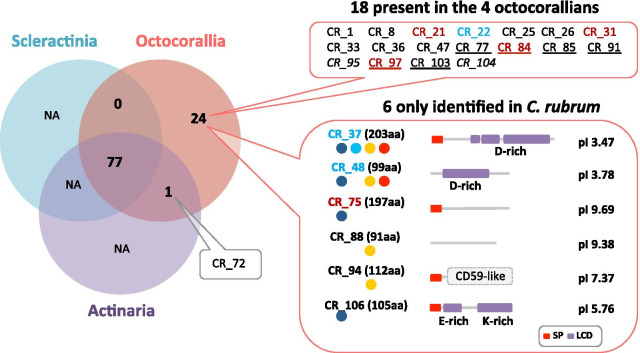
Conservation of the *C. rubrum* biomineral proteomes with other anthozoan species. Venn diagram depicts the conservation of the 102 *C. rubrum* OM proteins within anthozoan species. Among the 102 proteins, 77 share sequence similarity with hexacorallian (Scleractinia and Actiniaria) and octocorallian proteins, 1 (CR_72) is shared between Actiniaria and Octocorallia, and 24 are only found in octocorallians. Among the latter 24, 18 are shared in octocorallians and 6 are specific to *C. rubrum*. The 18 octocorallian proteins are listed and the 6 *C. rubrum* specific proteins are detailed as in Fig. 3b (pI: isoelectric point). Sequence names in blue correspond to proteins remarkable for their acidic pI and number of negative charges at pH = 8; names in red correspond to protein remarkable for their alkaline pI and number of positive charges at pH = 8 (see Additional file 3 sheet#2)

Among the striking features of the *C. rubrum* OM composition is the large number of proteins containing sugar-binding domains, most related to lectins (Fig. 2b; Additional file 3 sheet#2). In *C. rubrum*, 10 extracellular proteins contained carbohydrate-binding domains. CR_21, 22, 45, 47, 57 and 58 were found in both sclerites and axial skeleton OM whereas CR_65, 70, 85 and 97 were only found in the axial skeleton OM. BLAST search against NCBI_nr database revealed that CR_22 was very similar to proteins in the two octocorallians *Dendronephthya gigantea* and *Heliopora coerulea*, but not to other metazoan species (Fig. 5, Additional file 9). CR_22 was also similar to proteins in prokaryotes, with first hits in *Myxococcus* and *Actinoplanes* (Additional file 9). In order to conduct a phylogenetic analysis of the putative CR_22 homologs, we recurrently retrieved the first hits (regardless of the e-value) from different BLAST searches conducted against specific subdivisions of the metazoan tree (Additional file 9). PhyML analysis using the protein sequences from these BLAST results demonstrated that CR_22 had homologs in Octocorallia and Prokaryota, but not in other metazoan lineages, including Hexacorallia (Additional file 9). Importantly, CR_22 and its *D. gigantea* homolog are both encoded in their respective octocorallian genome; their genes contain introns with similar exon/intron organization (Additional file 9). Domain analyses indicated that CR_22 (and its homologs) encompasses a duplicate carbohydrate-binding domain family CBM25 [41], although with a low Pfam e-value. Thus, a plausible explanation is that the sugar-binding protein CR_22 could have been horizontally acquired through a prokaryote gene transfer early during Octocorallia evolution.

#### Carbonic anhydrases

Carbonic anhydrases (CAs) are metallo-enzymes that catalyze the reversible hydration of carbon dioxide into bicarbonate, the source of inorganic carbon for the precipitation of CaCO_3_. Here, CR_29, which corresponds to CruCA4 previously identified in the *C. rubrum* tissues [42], was identified with equivalent abundance in the proteome of SOM and IOM of the axial skeleton and the sclerites (emPAI: 1.15, 1.24, 0.64 and 0.71, respectively; Additional file 3 sheet#1).

### The *C. rubrum* OM compared with other cnidarians

In order to better understand the conservation of the *C. rubrum* OM proteins in regard to other anthozoans, the 102 *C. rubrum* OM proteins were BLAST searched against a selection of nucleotide databases from 3 anthozoan taxa (3 octocorallians, 3 actiniarians and 4 scleractinians; Additional file 10). Hits were retained for TBLASTN e-values below 1e-15 (no seg filter) and sequences were classified according to their taxon affiliation. Of the 102 OM proteins, 77 were found in at least one database of both subclasses (Octocorallia and Hexacorallia); 1 (CR_72) was found in octocorallians and actiniarians (not in scleractinians); 18 were shared only within the 4 octocorallian species and 6 were specific to *C. rubrum* (Fig. 5). Among the latter 6, structural analysis showed that Low Complexity Domains (LCDs) were dominant. LCDs have been described in the secreted OM of biominerals in different metazoan taxa [43–46]. We identified D-rich (aspartate-rich) LCD in CR_37 and CR_48, E-rich (glutamate-rich) and K-rich (lysine-rich) in CR_106. These D/E-rich domains involve a very acidic pI (Fig. 5, Additional file 3 sheet#2). Other LCDs such as G-rich have been identified in the OM of mollusk shell [47], but these domains were absent from the *C. rubrum* biomineral proteomes. Among the 24 proteins only identified in octocorallians, 9 were strongly alkaline (with pI > 9; CR_1, CR_21, CR_31, CR_47, CR_75, CR_84, CR_88, CR_91 and CR_97; Additional file 3 sheet#2), including the highly abundant scleritin (CR_1, conserved in the 3 tested octocorallians). Seven out of the 24 proteins (CR_75, 84, 85, 91, 97, 103 and 106; Additional file 3 sheet#2) were detected in the axial skeleton biomineral only.

**Fig. 6:**
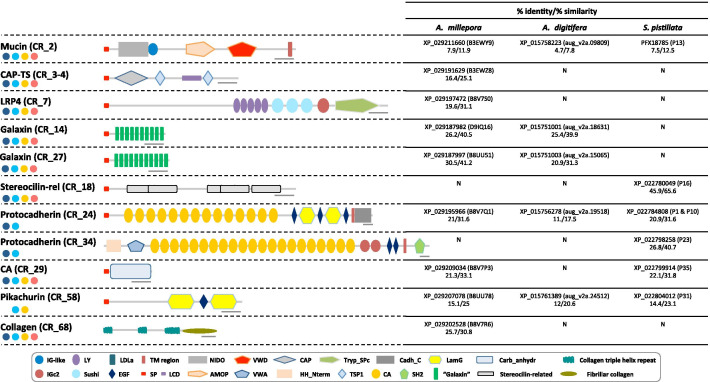
Schematic representation of eleven putatively conserved proteins identified in OM of the red coral and the scleractinian coral biominerals and their percentage of identity and similarity. Names, Proteome_ID and sequence description of the 11 *C. rubrum* proteins are indicated (left). Their presence in the different biomineral fractions is represented with circle as in Fig. 2b, and protein domains are described in the box. The 11 putative homologs were present in the published skeletome of 3 scleractinian species [16, 18, 21] and the OM of *C. rubrum* biominerals of our study (see Additional file 3 sheet#2). Percentage of identity and similarity between proteins of *C. rubrum* and proteins identified in the skeletome of the 3 other scleractinian species (Additional file 11) were calculated using https://www.bioinformatics.org/sms2/ident_sim.html and are indicated in the table (right; N: no putative homolog). For each scleractinian protein, GenBank accession number is indicated and published sequence name is given in brackets. Scale bars = 100 amino acids. *AMOP* adhesion-associated domain present in MUC4 and other proteins, *Cadh* cadherin repeats, *Cadh_C* cadherin_C, *CAP* cysteine-rich secretory proteins, antigen 5, and pathogenesis-related 1 protein, *CA* carbonic anhydrase, *EGF* epidermal growth factor, *HH_Nterm* hedgehog, *IG-like* immunoglobulin-like, *IGc2* immunoglobulin C-2 type, *LamG* laminin G, *LCD* low complexity domain, *LDLa* low-density lipoprotein receptor domain class A, *LY* low-density lipoprotein receptor YWTD domain, *NIDO* extracellular domain of unknown function in nidogen, *SH2* Src homology 2, *SP* signal peptide, *TM* transmembrane, *Tryp_SPc* trypsin-like serine protease, *TSP1* thrombospondin-1, *vWA* von Willebrand type A, *vWD* von Willebrand type D

With regards to the conservation of OM proteins between *C. rubrum* OM and stony corals, only 11 proteins (CR_2, 3–4, 7, 14, 18, 24, 27, 29, 34, 58 and 68) had homologs with constituents of the OM of the scleractinian biominerals (Fig. 6, Additional file 3 sheet#2 and Additional file 11). Within these 11 proteins, 2 were galaxin-like proteins, 1 was LRP and 2 were protocadherins. The two galaxin-like proteins (CR_14 and CR_27) exhibited 10 and 11 positions with a double di-cysteine motif that we identified using MEME discovery motif (Additional file 12). This motif was similar to the galaxin motif previously identified in the scleractinian coral, *A. millepora* [48]. In *C. rubrum*, these two galaxin-like proteins are present in both the axial skeleton and the sclerites. CR_7 (identified in both sclerites and axial skeleton) contains five low-density lipoprotein-receptor YWTD domains, as found in the mammalian LRP [49]. The mucin-like (CR_2) is one of the most abundant proteins. CR_3-4 is a prototype extracellular protein composed of the GAPR-1 (Golgi associated pathogenesis related-1) domain from the CAP superfamily followed by 2 TSP1 domains. Of note, we arguably accounted the cytoplasmic actin (CR_49) and the ubiquitin-60S ribosomal protein L40 (CR_67) as cellular contaminants, although they were also found in the OM of scleractinian corals.

## Discussion

Organic matrices (OMs) from biominerals are specialized ECMs that support the extracellular process of calcification. Among anthozoans, the OM of coral biominerals is widely studied in scleractinian (Hexacorallia) species [16, 18, 50–53], but poorly known in Octocorallia. Here, we studied the OM composition of the two high-magnesium calcite biominerals (the axial skeleton and the sclerites) produced by the octorallian species *Corallium rubrum*. This study aims to compare the protein repertoire (i) in both biominerals of *C. rubrum*, and (ii) with skeletomes of other anthozoan species. This new proteome provides novel understanding into the evolution of skeletomes in anthozoan biomineralization.

### The red coral axial skeleton and sclerite proteomes

The LC–MS/MS analysis of the axial skeleton and the sclerite OMs extracted from *C. rubrum* allowed us to identify 102 non-redundant proteins (Table 1). Organic matrices of both biominerals contain 52 common proteins, which may be due to the presence of the sclerites in the axial skeleton. In our study, scleritin, which was previously identified only in sclerites [13], is predominant in sclerites and in the axial skeleton. According to our estimates, sclerites only make up a minor component of the axial skeleton (3%). Given this, and the very high abundance of scleritin in the axial skeleton proteome, we suspect that scleritin is also secreted by the axial skeleton epithelium. The two biominerals also present specificities with 27 proteins only identified in the axial skeleton and 23 in the sclerites. Regarding SOM and IOM, the difference in solubility is unknown but may be explained by different degrees of post-translational modifications and/or increasing matrices complexity due to cross-linking with other types of molecules (e.g. polysaccharides). The present study is limiting by the unique peptide threshold (2 unique peptides per protein) and the fact that one replicate per sample was used for mass spectrometry analysis.

Organic matrix formation requires synthesis (secretion), assembly (e.g. cross-linking) and remodeling (e.g. digestion) of the various proteins forming the OM network [54, 55]. In the red coral proteome, we identified characteristic domains of proteins normally found in various ECMs (vWA, vWD, EGF-like, thrombospondin, sushi, reeler, fibrinogen, fibronectin and protocadherin) [56]. Several have matrix-remodeling function such as peptidases and peptidase inhibitors, which constitute a significant amount of the proteins fraction in various metazoan taxa [16, 57, 58]. The red coral skeletome possesses 7 peptidases including 4 matrix metallopeptidases (MMPs). MMPs are calcium-dependent zinc-containing endopeptidases with different substrate specificities central to the modeling of the OMs of mineralized tissues [59]. In mammals, MMP13 is known as the collagenase processing triple helical collagens (type I, II, and III among others) involved in bone remodeling and mineralization processes, whereas MMP12 digests elastin and a number of ECM molecules, and is also found in bone tissues [60–62]. The fact that each biomineral contains a different set of peptidases (serine peptidase and MMP13 in the axial skeleton, and cysteine peptidase and MMP12 in the sclerites) implies that their respective OM will be remodeled in different (specific) manners. Likewise, a prolyl 4-hydroxylase (P4H) homolog (CR_93) is found in the OM of the axial skeleton but not in the OM of the sclerites (Additional file 3 sheet#2). P4H is responsible for proline residues hydroxylation [63, 64], and its presence in the axial skeleton may reflect differences in collagen fibers post-translational modifications. The presence of proteins containing fibronectin (CR_84), protocadherin (CR_24 and CR_34) and peroxidase (CR_92) domains in the axial skeleton but not in the sclerites suggests functional differences between the set of OM proteins of each biomineral. In bilaterians, fibronectin binds to various other extracellular proteins [65, 66], protocadherin are trans-membrane adhesion proteins [67] and extracellular haem peroxidases have been involved in extracellular protein cross-linking [68, 69]. Altogether, these results suggest different matrix assembly properties of the axial skeleton OM compared with the OM of the sclerites, despite a similar composition in structural matrix proteins (such as collagen-like and agrin-like proteins).

### Evolutionary perspectives on coral calcification

The proteome composition of the red coral OMs exhibits numerous differences compared with stony coral skeletomes [16, 18, 21]. The most striking differences are the abundance of the structural collagen-like and agrin-like proteins, the cohort of protein carrying sugar-binding domains and the octocorallian specific OM proteins such as the predominant scleritin.

In *C. rubrum*, we identified 8 different collagen-like proteins in both biominerals (CR_5, 9, 17, 38, 39, 50, 54 and 56), 1 in the axial skeleton (CR_35) and 1 in the sclerites (CR_68). After synthesis of fibrillar collagens, the chains are extensively modified (e.g. in conserved triplet repeats G–X–Y, where Y is often proline, many proline residues are hydroxylated), an essential step for glycosylation and tertiary structuration of the collagen fibers [36]. The *C. rubrum* collagen-like sequences contain G–X–Y repeat motifs suggesting that they can assemble into fibers. Quantitatively, they represent a total 10–15% of the total amount of the OM proteins (Fig. 3c). This is highly contrasting with reef-building corals, where 1 collagen sequence was identified in *A. millepora* [16]. In gorgonian species, collagens have been found to be a major component of the sclerotized axis [70]. Thus, collagen-rich axis appears as a shared trait with gorgonians.

Then, we also identified 4 agrin-like proteins among which 3 are present in both *C. rubrum* biominerals. They represent candidate core-proteins for carrying glycoaminoglycans (GAGs), such as heparan sulfate or chondroitin sulfate. The presence of GAGs in the OM of *C. rubrum* has already been detected [19], but they were not identified in the OM of stony corals [16, 18]. In addition, the presence of an exostosin-3 homolog (CR_41), a protein responsible for the extension of heparan sulfate chains, corroborates the presence of GAGs in the red coral biominerals. The finding of CR_10-28-60 in the top 10 of highest abundant proteins and with equivalent relative abundance in the SOMax, IOMax, SOMsc and IOMsc (Additional file 6), points at this agrin-1 proteoglycan as an important structural component of the OM of both biominerals.

Lectins (carbohydrate-binding proteins) are widespread in animals [71–73], and have been described in mollusk shells, echinoderms skeletons and bird eggshells [74–76]. Polysaccharides make an important part of the skeletome of hexacorallian and octocorallian corals [16, 19]. The presence of at least 10 proteins containing sugar-binding domains suggests manifold possible interactions between proteins and polysaccharides. Among these, 6 (CR_21, 22, 45, 47, 57 and 58) are found in both biominerals of the red coral and 4 proteins (CR_65, 70, 85 and 97) are only identified in the axial skeleton OM, suggesting that the two matrices may also differ on the basis of specific sugar–protein interactions. Out of the 10 identified carbohydrate-binding proteins, 5 are only found in octocorallian species (CR_21, 22, 47, 85 and 97). Interestingly, CR_22 may have arisen through horizontal gene transfer (HGT) from a prokaryote to an octocoral ancestor (Fig. 5, Additional file 9). In cnidarians, HGTs have already been demonstrated for other protein families [77–79].

Based on sequence similarity (BLAST search with evalue < 1e-15), 24 *C. rubrum* OM proteins appear to be restricted to octocorallians. Among them, 18 are found in all 4 octocoral databases investigated, whereas 6 are only found in *C. rubrum*. Out of the 6 red coral-specific proteins, 3 proteins are LCD-rich. LCDs have been described in the secreted OM of biominerals in different metazoan taxa [43–46]. In previous proteomic studies on stony coral skeletons, these types of proteins were called Secreted Aspartic Acid-Rich Proteins (SAARPs [16]) and Coral Acidic Rich-Proteins (CARPs [18]). Such highly acidic macromolecules have a high affinity to positively charged ions such as calcium and may thus play a role in CaCO_3_ formation [52, 80, 81]. On the other hand, 9 of the 24 octocorallian-specific proteins are strongly alkaline, including scleritin (CR_1). Scleritin was first identified as a sclerite specific protein [13].

Stony coral skeletons are composed of aragonite, whereas red coral skeletons are composed of calcite [7, 15–18]. Our proteomic study demonstrates that the underlying skeletomes of stony and red corals also differ, which extend a recent proteomic study realized on the sclerites of 3 octocoral and 1 scleractinian species [82]. Additionally, our study reveals similarities in the axis composition of alcyonaceans in the form of a collagen-rich matrix. Thus, we propose that the red coral axial skeleton biomineralization is the evolutionary result of the calcification of the gorgonian axis. This hypothesis is also supported by other evidence of axis mineralization in alcyonaceans. For instance, in the bamboo coral (*Keratoisis* genus) the axis is made of alternating structures of gorgonin (nodes) and calcite (internodes) [83]. In the sea rod *Plexaurella nutans*, calcite loculi precipitate in the axis [84]. However, only red corals produce a fully calcified axis. According to estimated phylogenetic divergence between Octocorallia and Hexacorallia (Precambrian) [53, 85, 86], calcification would have been acquired independently in these two subclasses during anthozoan evolution.

### Calcification toolkit in anthozoans

Among the 77 proteins shared between octocorallians, actiniarians and hexacorallians, only 11 are present in the OM of both stony and red corals biominerals (Fig. 6, Additional file 3 sheet#2). The 11 proteins are distributed in 9 protein families (1 mucin, 1 CAP-like protein, 2 galaxin-like proteins, 2 protocadherins, 1 LRP, 1 CA, 1 collagen-like protein, 1 pikachurin-like protein and 1 unknown protein).

Mucins are a family of high molecular weight, heavily glycosylated proteins (glycol-conjugates) produced by epithelial tissues in most animals. In addition to their role in vertebrate mucus formation [87], they also participate in the calcification of mollusk shells, echinoderm skeletons and vertebrate bones [88–90]. Their conservation into the process of anthozoan calcification pinpoints them as major players into the mechanism of calcification in metazoans. In the CAP-like protein (CR_3-4), two domains were identified, GAPR-1 and TSP1. The GAPR-1 domain found has been shown to form amyloid-like fibrils in the presence of acidic phospholipids [91]. TSP1 repeats can physically interact with a variety of ligands, including structural components of the ECM, other matricellular proteins, growth factors, and proteases [92, 93]. Although a structural role may be envisaged, the function of CR_3-4 remains to be clarified. LRP protein was also previously identified in the skeletome of scleractinian corals [21] and echinoderms [94]. LRP4 is expressed in chondrocytes and promotes cell-autonomous cartilage growth in association with agrins [95]. However, LRPs are cell-surface (co)receptors involved in intracellular signal transduction and virtually nothing is known on the signaling control of calcification in anthozoans. Likewise, protocadherins (CR_24 and CR_34) are also known as trans-membrane proteins involved in cell–cell adhesion. The conserved function of these cell surface macromolecules in the calcification process remains to be deciphered.

Two galaxin-like proteins (CR_14 and CR_27) are identified in both the axial skeleton and the sclerites of the red coral. Galaxin was first identified in the exoskeleton of the scleractinian coral *Galaxea fascicularis* and was described as a tandem repeat structure with a di-cysteine motif fixed at nine positions [96]. Since this discovery, galaxin homologs have been observed in the exoskeleton of other scleractinian species [16, 21, 97]. Galaxin is associated with the developmental onset of calcification after larval stage in *A. millepora* [48]. Galaxin-like proteins were first suggested to be cnidarian-specific proteins [48, 96] until their discovery in mollusks and annelids [98–100]. In the squid *Euprymna scolopes,* a galaxin-like protein called EsGal1, was identified as an antimicrobial protein involved in the selection and modulation of growth of its symbiont, the Gram-negative bacterium *Vibrio fischeri* [98]. The high conservation of this motif in the different taxa suggests that this di-cysteine motif might play an essential role.

In metazoans, carbonic anhydrases (CAs) belong to multigenic family and are widely known to be involved in biomineralization in diverse metazoans such as sponge spicules [101, 102], mollusk shells [103, 104], sea urchin skeleton [105] and bird eggshells [106, 107], as well as in stony corals [16, 18, 50, 51, 108, 109]. In *C. rubrum*, 6 CAs were previously identified (CruCA1 to 6) and based on tissue expression profiles, CruCA4 was proposed to be the CA involved in the biomineralization process [25]. We now identified CruCA4 (CR_29) in both the axial skeleton and the sclerites, which supports its possible role in the biomineralization of the red coral. With regards to phylogeny, CruCA4 likely represents a duplicated copy of CruCA6, which is not phylogenetically related to other coral CAs involved in calcification [42]. Previous studies emphasize that the involvement of CAs in calcification likely represents a functional convergence of evolution of the calcification process [42, 45]. Even with some similar proteins in the calcification toolkit of stony and red corals, the independent acquisition of calcification is the most credible scenario. The presence of similar/homologous domains/proteins in the coral skeletomes could be the result of independent gene recruitment in anthozoan taxa.

## Conclusions

This work is the first proteomic study of the organic matrix of the precious red coral (*C. rubrum*) biominerals. We report a total of 102 identified proteins, scleritin being the most abundant protein in the OM of both the sclerites and the axial skeleton. We noticed the abundant presence of collagen-like proteins (9 in each biomineral on 10 identified in our study), and highlighted 4 agrin-like proteins (3 in both biominerals and 1 more in the sclerites), which are ECM glycoproteins. These reuslts support the view that the calcified axial skeleton of the red coral evolved from the horny skeleton of gorgonians. Comparative analysis of the protein composition between the two biominerals pointed to differences in the processing (assembly/remodeling) of the OM rather than its structural composition. Indeed, specific enzymes (e.g. endopeptidases) and proteins harboring distinct sugar-binding domains are particular to each biomineral. Finally, we identified 18 octocorallian-specific proteins (of which 1 is a putative HGT from prokaryotes) and 6 unique *C. rubrum* novelties. Compared with stony corals, only few proteins were conserved (e.g. mucin, carbonic anhydrase, galaxins and protocadherins). The presence of these proteins in both octocorallian and scleractinian biomineralizations potentially represents a convergent co-option of calcification toolkit in corals.

## Methods

### Biological material

*Corallium rubrum* colonies were collected by the IMBE of Marseille in the Mediterranean Sea near the Marseille coast (Plane Island, Gulf of Lion: 43° 11.190′ N, 5° 23.470′ E), at a depth of 10 m. The collection was authorized by the Maritime Prefect of Bouches-du-Rhône (France) and the sampling did not involve permanent injuries, i.e. colonies recovered within two weeks after cutting. Colonies were transferred to the Centre Scientifique de Monaco to be cultured in open-circuit seawater aquaria supplied with Mediterranean seawater at a temperature of 18 °C ± 2 °C and were fed five times per week with frozen red plankton, rotifers and living artemia.

### Extraction procedure of organic matrices

Axial skeletons and sclerites of red corals were isolated from living tissue by incubating branches in 10% hypochlorite under slow stirring and then rinsed five times in milli-Q water. Samples were subsequently dried at 60 °C. The axial skeleton and the sclerites were separated and washed 3 times in 10% hypochlorite for 24 h under agitation. Sclerites present in the axis cannot be separated as they are cemented in the medulla of the axial skeleton. Axis skeleton samples were pooled and crushed in liquid nitrogen for 5 min to obtain a homogeneous powder. The powder of axis and the non-crushed sclerites were cleaned in 10% hypochlorite overnight at 4 °C under stirring, then rinsed 8 times with milli-Q water and dried according to a published protocol [27]. The powder from pooled axis (10 g) and the pooled sclerites (5 g) were decalcified using 4% acetic acid for 24 h at 4 °C under stirring. Decalcified samples were centrifuged at 3500 g for 10 min at 4 °C to separate the supernatant containing the acidic-soluble fraction (soluble organic matrix—SOM) from the pellet containing the acidic-insoluble fraction (insoluble organic matrix—IOM). To remove acidic solutions, each SOM sample was diafiltered in a 3 kDa ultrafiltration cell (Amicon, Millipore) by centrifugation at 3500 g for 20 min at 4 °C. After the first passage of the decalcification solution, milli-Q water was added and centrifuged again. In total eight cycles of centrifugation-resuspension were performed. The pellets containing the IOMs were resuspended in milli-Q water, centrifuged and the supernatants discarded.

### Electrophoresis and trypsin treatment of organic matrices

A total of 100 µg of SOM, and non-quantified IOM, extracted from pooled axial skeletons and sclerites (called SOMax, IOMax and SOMsc, IOMsc), were denatured in Laemmli buffer for 5 min at 95 °C. Proteins were loaded on a 12% polyacrylamide SDS-PAGE electrophoretic gel for rapid run (15 min) in order to obtain one single band. Gels were colored using Coomassie blue staining and rinsed several times with milli-Q water. Gels were then packed in sterile bags with 1% acetic acid until the trypsin treatment. The single bands were excised from the gels and cut into pieces. Reduction was performed using 10 mM dithiothreitol (DTT) in 100 mM NH_4_HCO_3_, 30 min at 37 °C. Gels were then cooled at room temperature and the excess DTT was removed. Alkylation was done with 55 mM Iodoacetamide in 100 mM NH_4_HCO_3_ for 1 h at room temperature, with occasional vortexing. Gels were washed with 100 mM NH_4_HCO_3_, dehydrated in 100 μl of acetonitrile and re-swelled 100 mM NH_4_HCO_3_ for 5 min. Gels were dehydrated once again with 100 μl of acetonitrile and dried in vacuum centrifuge (Savant Speedvac). For digestion, gels were firstly incubated in digestion solution containing 12.5 ng/μl of trypsin (Sequencing Grade Modified Trypsin, Promega, ref. V5111) in 50 mM NH_4_HCO_3_ during 30 min at 4 °C. Then, the excess of trypsin was removed and 50 mM NH_4_HCO_3_ was added on gels and trypsin in gel digested proteins overnight at 37 °C. After digestion, samples were cooled at room temperature and centrifuged 10 min at 6000 rpm. The supernatant was saved in a new microtube. Gels were then successively treated with 20 mM NH_4_HCO_3_ and 5% formic acid in 50% aqueous acetonitrile, centrifuge 5 to 10 min at 6000 rpm and supernatants were saved. The three supernatants/samples were finally pooled and dried in vacuum centrifuge.

### NanoLC-MS/MS analysis

NanoLC-MS/MS analysis was performed once on each sample (SOMax, IOMax, SOMsc and IOMsc) using an on-line system consisting of a nano-pump UltiMate™ 3000 UHPLC binary HPLC system (Dionex, ThermoFisher) coupled to a Q-Exactive HF mass spectrometer (ThermoFisher, Germany). Peptides were resuspended in 20 μl of sample buffer (3% acetonitrile, 0.1% formic acid) and 2 μl was injected into a pre-column 300 µm × 5 mm (Acclaim PepMap, 5 µm particle size). After loading, peptides were eluted to an Acclaim PepMap100 C18 capillary column (75 µm × 15 cm, 100 Å, 3 μm particle sizes). Peptides were eluted into the MS, at a flow rate of 300 nl/min using a 40 min gradient from 5 to 40% mobile phase B. Mobile phase A was 0.1% formic acid in H_2_O and mobile phase B was 80% acetonitrile and 0.1% formic acid. The mass spectrometer was operated in positive and data-dependent mode, with a single MS scan (m/z 350–1400 at 60,000 resolution (at m/z 200) in a profile mode) followed by MS/MS scans on the 10 most intense ions at 15,000 resolution. Ions selected for MS/MS scan were fragmented using higher energy collision dissociation (HCD) at normalized collision energy of 28% and using an isolation window of m/z 1.8.

The raw mass spectrometric data have been deposited to PRIDE via the ProteomeXchange Consortium (https://www.ebi.ac.uk/pride/archive/) with the project accession PXD020332.

### Protein identification and in silico characterization

The RAW files from Q-Exactive HF were converted into Mascot generic format (mgf) files using Proteome Discoverer v1.4 (Thermo Scientific). These files were submitted to Mascot v2.3 (Matrix Sciences Ltd, United Kingdom) for database search against the red coral transcriptome database of the Centre Scientifique de Monaco (CrubmRNA.fasta; data available at http://data.centrescientifique.mc/CSMdata-crubrum_data.html). The mass tolerance was set to 20 ppm for precursors, and 0.5 Da for the MS/MS fragment ion. The fixed modifications were set to carbamidomethyl and variable modifications were set to oxidation at methionine. The Mascot result files were processed using Scaffold v4.8.3 (Proteome Software Inc. USA; Additional file 13) software using the Prophet algorithm for validation of peptide and protein identifications with a threshold of 95% and 99%, respectively, and a minimum of two peptides in one of the 4 fractions for the identification of one protein. The relative abundance of identified proteins in each organic matrix fraction was estimated by calculating the exponentially modified protein abundance index (emPAI) using Scaffold v4.8.3. In order to compare the abundance of proteins in each condition, the weighted spectra (WS) value was also calculated as the number of MS/MS spectra for each protein (Additional file 3 sheet#1).

Identified proteins using Mascot were cross-checked against the published *C. rubrum* transcriptome [32], which used a different assembly strategy. This allowed us to merge several sequences from the CrubmRNA.fasta database into longer cDNA sequences (Additional file 2). Cellular localization was determined from the prediction of signal peptide (SignalP; http://www.cbs.dtu.dk/), transmembrane domain (TMHMM; http://www.cbs.dtu.dk/) and GPI-anchor (assigned GPI if both in predGPI and in GPISOM; http://gpcr.biocomp.unibo.it/predgpi/pred.htm: http://gpi.unibe.ch/). Sequence annotation was carried out using Blast2GO pipeline (B2G: https://www.blast2go.com/) using BLASTP against refseq (Eval > 1E-15) for homology search. ProtParam (http://web.expasy.org/protparam/) was used to determine in silico molecular weight and pI of proteins, net charge pH = 8 was calculated with PROTEIN CALCULATOR v3.4 (http://protcalc.sourceforge.net/), Low Complexity Region were determined using segmasker (SEG: http://mendel.imp.ac.at/METHODS/seg.server.html), and InterProScan (B2G) and SMART (http://smart.embl-heidelberg.de) were used for domain predictions (Additional file 3 sheet #2).

### Multiple sequence alignment and phylogenetic analysis

Multiple sequence alignments for collagen-like and galaxin-like proteins were performed using Mafft (https://www.ebi.ac.uk/Tools/msa/mafft).

For CR_22 phylogenetic analysis, we did independent BLAST search against different taxonomic subdivisions of the NCBI_nr protein database: Cnidaria/Octocorallia, Cnidaria/Hexacorallia, Cnidaria/Hydrozoa, Deuterostomia, Protostomia, Placozoa, Porifira), as well as the Prokaryota subdivision. The first hit of each search as retrieved and subjected to clustalΩ MSA. Best-fit models of amino-acid replacement were calculated using ProtTest v3.4 [110]. Phylogenetic tree was reconstructed using PhyML v3.0 [111]. The tree was build using VT model with a gamma shape of 3.48, and likelihood topology was estimated by SPR (subtree pruning and regrafting) moves.

For BLAST homology search within anthozoan, local BLAST (2.2.27 +) was used. Species-specific databases were merged and interrogated as detailed in main text.

Scleractinian OM homologs to *C. rubrum* OM proteins were identified based on the published domain compositions [16, 18, 21] in addition to BLAST homology searches described above. We next retrieved the full-length sequences corresponding to the published sequences for *A. millepora*, *A. digitifera* and *S. pistillata* (which some were partial). Then, each set of *C. rubrum* protein and their respective putative homologs were aligned using Mafft. Finally, the percentages of amino-acid identity and similarity for each MSA were calculated using “Ident and Sim” (https://www.bioinformatics.org/sms2/ident_sim).

## Supplementary Information

**Additional file 1:** List of the 34 identified proteins in *Corallium rubrum* biominerals that are distributed into 9 protein families.

**Additional file 2:** Genomic and transcriptomic sequences corresponding to the 107 proteins initially identified in *Corallium rubrum* biominerals. The final sequences for CR3_4, CR_10_28_60, and CR_62_76_82 are also listed.

**Additional file 3:** Table of details for the 102 identified proteins in *C. rubrum* biominerals. For each identified protein in each OM fraction, unique peptide count, spectrum count, percentage of spectra, protein probability, percent protein coverage, exponentially modified protein abundance index (emPAI) and weighted spectra (WS) values are reported in sheet#1. Amino acid length, pI, charge at pH = 8, molecular weight (MW in Dalton Da), low complexity region, InterProscan protein signatures, description, subcellular (SubCell) localization prediction, BLAST (NCBI_ref-seq) result with first hit species and taxonomy, conservation in other anthozoan (BLAST e-val > 1e-15) and in scleractinian OM are listed in sheet#2. SOMax: Soluble Organic Matrix of the axial skeleton (dark blue), IOMax: Insoluble Organic Matrix of the axial skeleton (blue), SOMsc: Soluble Organic Matrix of the sclerites (yellow), IOMsc: Insoluble Organic Matrix of the sclerites (red). Empty circles: 1 unique peptide count.

**Additional file 4:** Relative estimation of the proportion of sclerites into the axial skeleton using 7 published axial skeleton cross-sections [7, 33]. a: annular part; m: medullar part. Medullar and annular areas were drawn by hand after the published pictures with Photoshop®. The resulting png files were analyzed using ImageJ. The relative calculated areas are shown in the table.

**Additional file 5:** Venn diagram distribution of the 102 identified proteins using 1 unique peptide threshold, in the IOM and SOM of the axial skeleton and the sclerites of *C. rubrum*.

**Additional file 6:** List of the 10 most abundant proteins in each *C. rubrum* sample. a. Table of the 10 most abundant proteins in each OM biomineral fraction according to their exponentially modified protein abundance index (emPAI) value. Proteins highlighted in grey are in the top 10 of each sample, proteins highlighted in red are in the top 10 of SOM and IOM of the axial skeleton and proteins highlighted in blue are in the top 10 of SOM and IOM of the sclerites. b. Relative abundance in weighted spectra of the ten most abundant proteins in the red coral biominerals. SOMax: Soluble Organic Matrix of the axial skeleton (dark blue bars), IOMax: Insoluble Organic Matrix of the axial skeleton (blue bars), SOMsc: Soluble Organic Matrix of the sclerites (yellow bars), IOMsc: Insoluble Organic Matrix of the sclerites (red bars).

**Additional file 7:** Multiple sequence alignment of the 10 collagen-like proteins identified in the proteome of the *C. rubrum* biominerals. Multiple alignment was carried out using Multalin (http://multalin.toulouse.inra.fr/multalin/multalin.html) and edited using JalView. Note the G-X–Y triplet repeats (glycine in blue, proline in red) characteristic of collagens.

**Additional file 8:** Wrongly assigned collagen sequences in *C. rubrum* (top) and *S. pistillata* (bottom). Both in our analysis and in Drake and coworkers [18], the *C. rubrum* CR_98 and the *S. pistillata* XP_022783415 (P14) and AGG36343 (P18) sequences were wrongly assigned as “collagen”, probably because they are containing a von Willebrand factor type A domain (vWA) very homologous to another vWA domain sequence from another organism, which is there associated to a collagen protein sequence. Hence, by transitivity, the coral sequences were automatically annotated as “collagen”, although not being a collagen. Likewise, the *C. rubrum* CR_6 was also automatically annotated as “collagen”, as it contains a ColF1 domain. However, since the rest of the sequence is fibrinogen-related and does not contain G-X-Y triplets, CR_6 is assigned as “fibrillar collagen”, but not considered in the set of the collagens. COLFI: fibrillar collagen C-terminal domain; FA58C: coagulation factor 5/8 C-terminal domain; FBG: fibrinogen-related domain; TSP1: thrombonspondin-1.

**Additional file 9:** CR_22 as a gene horizontally transferred from unicellular organisms. a. Results of the BLAST search in the different taxa in NCBI database (Porifera, Placozoa, Cnidaria/Octocorallia, Cnidaria/Hexacorallia, Cnidaria/Hydrozoa, Protostomia, Deuterostomia and Bacteria). b. Multiple alignment of CR_22 with its 2 octocorallian homologs (HeCoe: *Heliopora coerulea*; DeGig: *Dendronephthya gigantea*) and 2 prokaryotes (MyXan: *Myxococcus xanthus;* AcXin: *Actinoplanes xinjiangensis*). The two carbohydrate-binding domains CBM25 (Pfam) of CR_22 are framed in red box. c. Genomic exon (green squares)/intron (dashed blue lines) structure of the CR_22 and DeGig_CBM25 gene coding sequences. Numbers correspond to the introns’ sizes in base pair. d. Phylogenetic tree (PhyML) of CR_22 homologs and proteins corresponding to first BLAST hit from a.

**Additional file 10:** List of the six anthozoan species used for the comparison with *Corallium rubrum* biominerals proteome. Nucleotide databases are indicated for each species.

**Additional file 11:** List of putative homologs between *Corallium rubrum* biomineral proteomes and skeletomes of three scleractinian species *Acropora millepora*, *Acropora digitifera* and *Styllophora pistilata* [16, 18, 21].

**Additional file 12:** Galaxin-like proteins. The published protein sequences for galaxin homologs from *Acropora millepora, Galaxea fascicularis*, *Stylophora pistillata* and *Euprymna scolopes* [16, 48, 96–98], as well as the present *Corallium rubrum* CR_14 and CR_27 were submitted to the MEME discovery motif and the MAST motif scanning programs (http://meme-suite.org/). The motifs is a double di-cysteine motif similar to galaxin motif found by Reyes-Bermundez and colleagues [48], and is shown as logo on top. On the listed fasta sequences, this double di-cysteine motif, or galaxin motif, is highlighted in yellow in each sequence. The signal peptide sequence (http://www.cbs.dtu.dk/services/SignalP-5.0/) is in italic and underlined.

**Additional file 13:** List of proteins and peptides identified in the SOMs and IOMs of the axial skeleton and the sclerites of *Corallium rubrum*. For each identified protein, identified peptides are listed. For each peptide, amino acid sequence, spectra and Mascot information are listed. SOMax: soluble organic matrix of the axial skeleton (sheet#1); IOMax: insoluble organic matrix of the axial skeleton (sheet#2); SOMsc: soluble organic matrix of the sclerites (sheet#3); IOMsc: insoluble organic matrix of the sclerites (sheet#4).

## Data Availability

The transcriptome of *Corallium rubrum* (CrubmRNA.fasta) that is used for analyses in the present study is available in the public database of the Centre Scientifique de Monaco (http://data.centrescientifique.mc/CSMdata-crubrum_data.html). The dataset supporting the conclusions of this article is included within the article and its Additional files 2 and 13. The raw mass spectrometric data have been deposited to PRIDE via the ProteomeXchange Consortium (https://www.ebi.ac.uk/pride/archive/) with the project accession PXD020332.

## Abbreviations

CaCO_3_: Calcium carbonate
DTT: Dithiothreitol
ECM: Extracellular matrix
emPAI: Exponentially modified protein abundance index
GAG: Glycosaminoglycan
HGT: Horizontal gene transfer
IOM: Insoluble organic matrix
LRP: Low-density lipoprotein receptor-related protein
MMP: Matrix metalloproteinase
MSA: Multiple sequence alignment
NanoLC-MS/MS: Nano-liquid chromatography-tandem mass spectrometry
SOM: Soluble organic matrix
OM: Organic matrix
vWA: Von Willebrand factor type A
WS: Weighted spectral count
