# Supplementary material for: The skeletome of the red coral Corallium rubrum indicates an independent evolution of biomineralization process in octocorals
Source: BMC Ecol Evol. 2021 Jan 11;21:1. doi: 10.1186/s12862-020-01734-0 (PMC7853314; doi:10.1186/s12862-020-01734-0)
Supplement: Supplementary file 4 — Additional file 4: Relative estimation of the proportion of sclerites into the axial skeleton using 7 published axial skeleton cross-sections [7, 33]. a: annular part; m: medullar part. Medullar and annular areas were drawn by hand after the published pictures with Photoshop®. The resulting png files were analyzed using ImageJ. The relative calculated areas are shown in the table. [file 12862_2020_1734_MOESM4_ESM.pdf]

Additional file 4

Vielzeuf *et al.*, 2008_Fig2

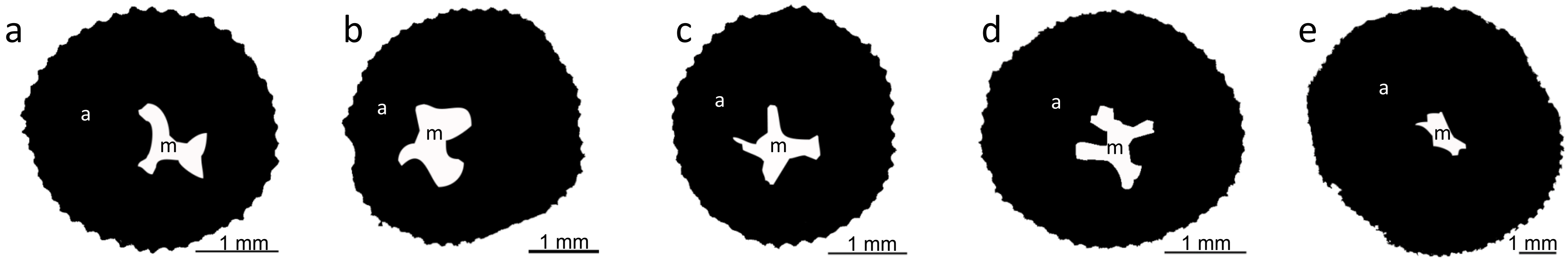

Chaabane *et al.*, 2019_Fig2

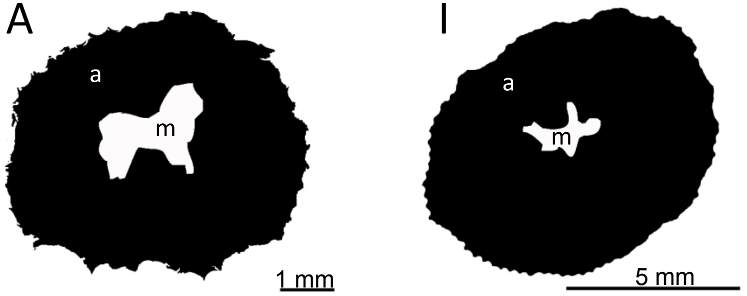

Photograph	Area (m²)	Med/Ann
Vielzeuf2008_a_Medulla	5975	**4.88%**
Vielzeuf2008_a_Annula	128515	
Vielzeuf2008_b_Medulla	7840	**7.96%**
Vielzeuf2008_b_Annula	106388	
Vielzeuf2008_c_Medulla	6470	**5.65%**
Vielzeuf2008_c_Annula	120973	
Vielzeuf2008_d_Medulla	6384	**5.10%**
Vielzeuf2008_d_Annula	131465	
Vielzeuf2008_e_Medulla	2047	**1.80%**
Vielzeuf2008_e_Annula	115770	
Chaabane2019_A_Medulla	20140	**9.66%**
Chaabane2019_A_Annula	228524	
Chaabane2019_I_Medulla	6387	**2.55%**
Chaabane2019_I_Annula	257162	
	Mean	**5.37%**
	SEM	**1.05%**
