# Supplementary figures and images for: The skeletome of the red coral Corallium rubrum indicates an independent evolution of biomineralization process in octocorals
Source: BMC Ecol Evol. 2021 Jan 11;21:1. doi: 10.1186/s12862-020-01734-0 (PMC7853314; doi:10.1186/s12862-020-01734-0)

Additional file 5

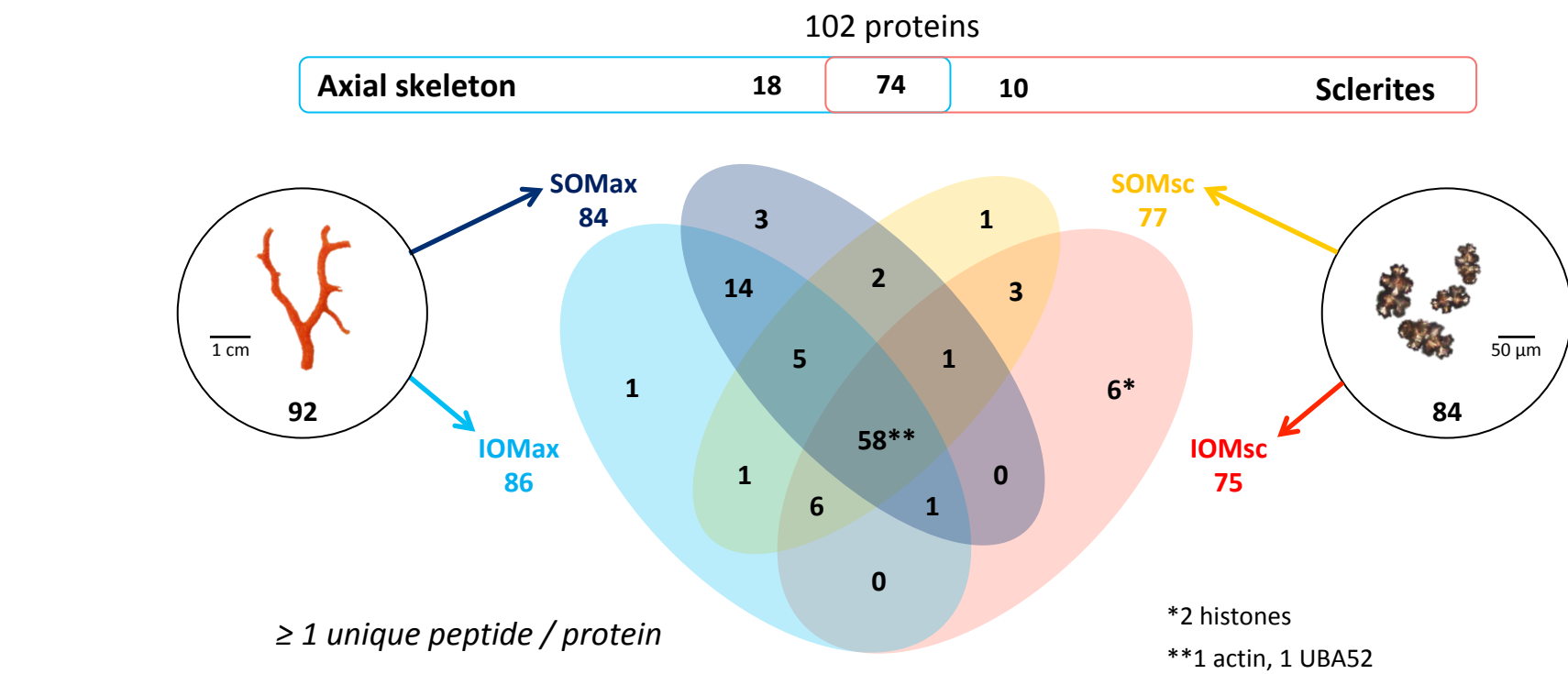

Supplement: Supplementary file 5 — Additional file 5: Venn diagram distribution of the 102 identified proteins using 1 unique peptide threshold, in the IOM and SOM of the axial skeleton and the sclerites of C. rubrum. [file 12862_2020_1734_MOESM5_ESM.pdf]

Non-collagenous organic matrix proteins

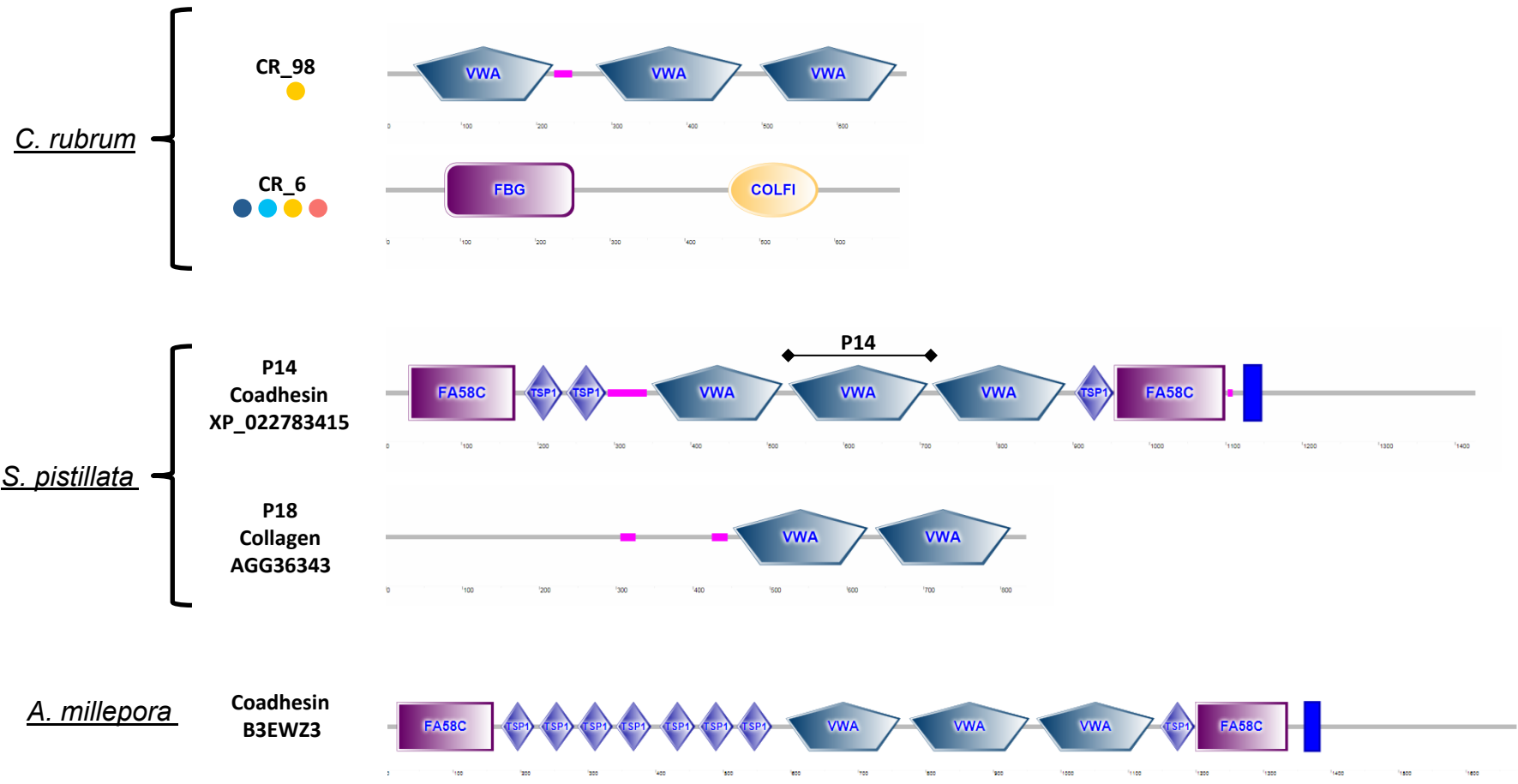

Supplement: Supplementary file 8 — Additional file 8: Wrongly assigned collagen sequences in C. rubrum (top) and S. pistillata (bottom). Both in our analysis and in Drake and coworkers [18], the C. rubrum CR_98 and the S. pistillata XP_022783415 (P14) and AGG36343 (P18) sequences were wrongly assigned as “collagen”, probably because they are containing a von Willebrand factor type A domain (vWA) very homologous to another vWA domain sequence from another organism, which is there associated to a collagen protein sequence. Hence, by transitivity, the coral sequences were automatically annotated as “collagen”, although not being a collagen. Likewise, the C. rubrum CR_6 was also automatically annotated as “collagen”, as it contains a ColF1 domain. However, since the rest of the sequence is fibrinogen-related and does not contain G-X-Y triplets, CR_6 is assigned as “fibrillar collagen”, but not considered in the set of the collagens. COLFI: fibrillar collagen C-terminal domain; FA58C: coagulation factor 5/8 C-terminal domain; FBG: fibrinogen-related domain; TSP1: thrombonspondin-1. [file 12862_2020_1734_MOESM8_ESM.pdf]
