# Supplementary material for: The skeletome of the red coral Corallium rubrum indicates an independent evolution of biomineralization process in octocorals
Source: BMC Ecol Evol. 2021 Jan 11;21:1. doi: 10.1186/s12862-020-01734-0 (PMC7853314; doi:10.1186/s12862-020-01734-0)

Additional file 6

a

CR_n	emPAI SOM Ax	CR_n	emPAI IOM Ax	CR_n	emPAI SOM Sc	CR_n	emPAI IOM Sc
CR_1	32.011	CR_1	30.225	CR_1	71.871	CR_1	66.78
CR_3_4	7.2949	CR_3_4	8.9802	CR_3_4	4.4624	CR_3_4	5.1672
CR_8	3.7254	CR_5	4.3196	CR_5	2.1084	CR_6	2.3425
CR_5	2.9257	CR_8	3.2866	CR_6	1.7873	CR_5	2.334
CR_14	2.8288	CR_10_28_60	2.61144	CR_48	1.5899	CR_10_28_60	2.00068
CR_10_28_60	2.55556	CR_12	2.5931	CR_2	1.5399	CR_55	1.9737
CR_22	2.3529	CR_33	2.5602	CR_10_28_60	1.48017	CR_2	1.1644
CR_48	2.1927	CR_9	2.2777	CR_25	1.2092	CR_99	1.1325
CR_26	1.9963	CR_16	1.6715	CR_14	0.96766	CR_48	1.1049
CR_9	1.9856	CR_2	1.4888	CR_8	0.93538	CR_22	1.0103

b

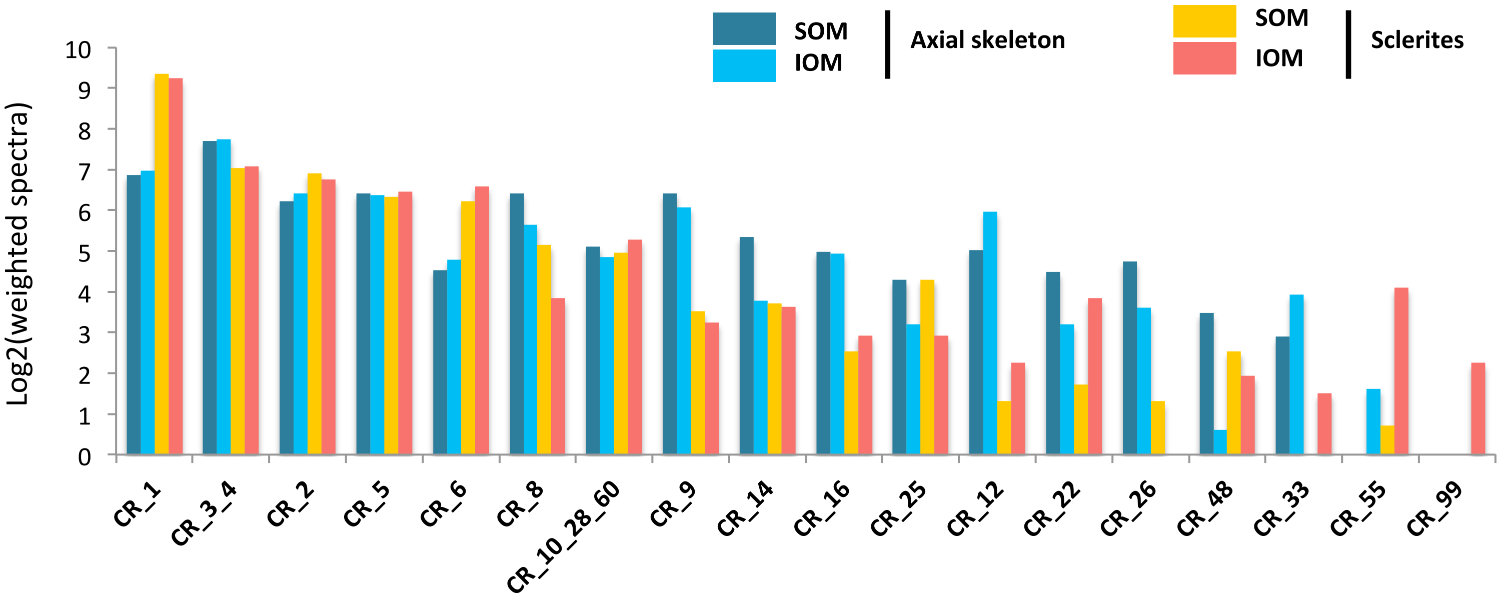

Supplement: Supplementary file 6 — Additional file 6: List of the 10 most abundant proteins in each C. rubrum sample. a. Table of the 10 most abundant proteins in each OM biomineral fraction according to their exponentially modified protein abundance index (emPAI) value. Proteins highlighted in grey are in the top 10 of each sample, proteins highlighted in red are in the top 10 of SOM and IOM of the axial skeleton and proteins highlighted in blue are in the top 10 of SOM and IOM of the sclerites. b. Relative abundance in weighted spectra of the ten most abundant proteins in the red coral biominerals. SOMax: Soluble Organic Matrix of the axial skeleton (dark blue bars), IOMax: Insoluble Organic Matrix of the axial skeleton (blue bars), SOMsc: Soluble Organic Matrix of the sclerites (yellow bars), IOMsc: Insoluble Organic Matrix of the sclerites (red bars). [file 12862_2020_1734_MOESM6_ESM.pdf]
