# Supplementary material for: The skeletome of the red coral Corallium rubrum indicates an independent evolution of biomineralization process in octocorals
Source: BMC Ecol Evol. 2021 Jan 11;21:1. doi: 10.1186/s12862-020-01734-0 (PMC7853314; doi:10.1186/s12862-020-01734-0)

Additional file 7

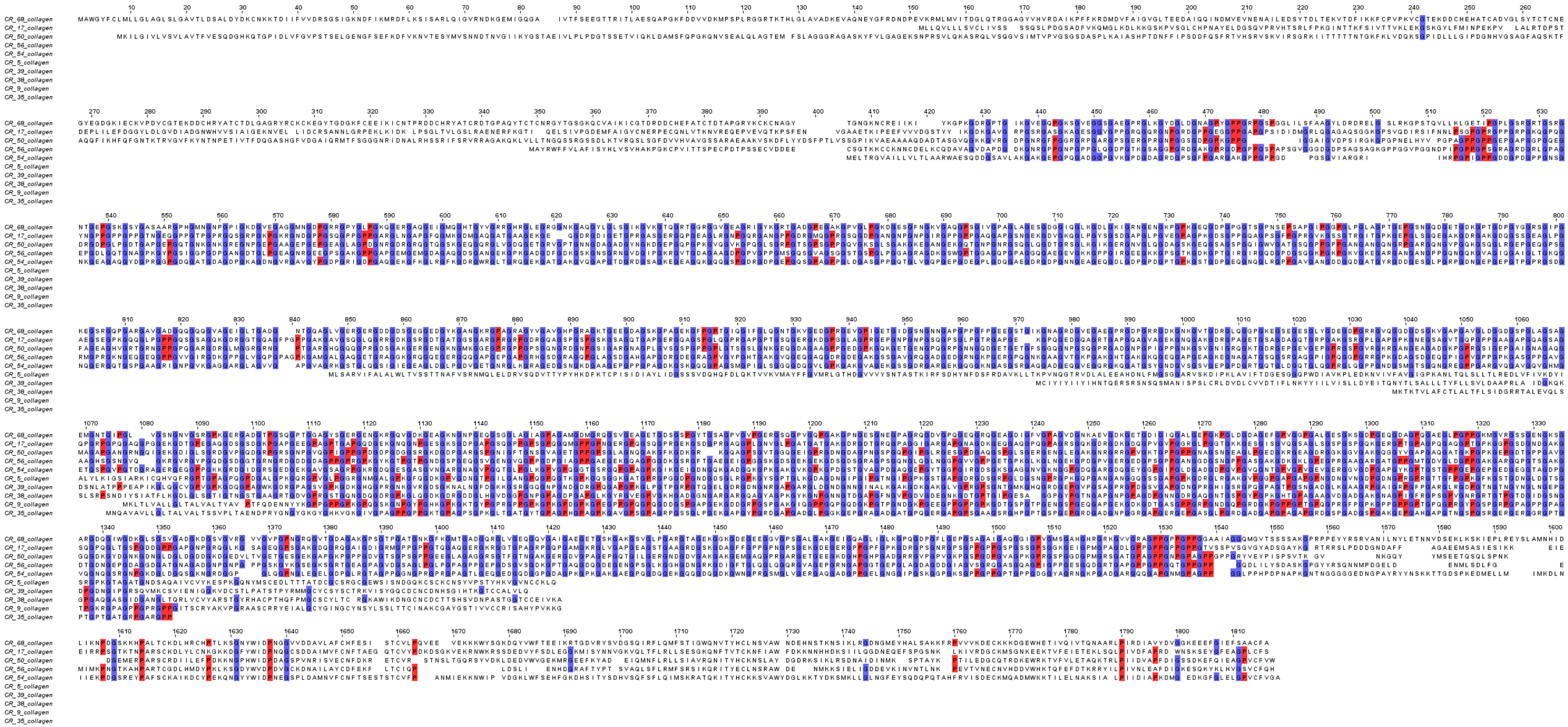

Supplement: Supplementary file 7 — Additional file 7: Multiple sequence alignment of the 10 collagen-like proteins identified in the proteome of the C. rubrum biominerals. Multiple alignment was carried out using Multalin (http://multalin.toulouse.inra.fr/multalin/multalin.html) and edited using JalView. Note the G-X–Y triplet repeats (glycine in blue, proline in red) characteristic of collagens. [file 12862_2020_1734_MOESM7_ESM.pdf]
