# Supplementary material for: The skeletome of the red coral Corallium rubrum indicates an independent evolution of biomineralization process in octocorals
Source: BMC Ecol Evol. 2021 Jan 11;21:1. doi: 10.1186/s12862-020-01734-0 (PMC7853314; doi:10.1186/s12862-020-01734-0)

Additional file 9

a

CR_22	phylum	Cnidaria					Deuterostomia	Protostomia	Placozoa	Porifera	Bacteria	
	database: nr/	Octocorallia		Hexacorallia		Hydrozoa	Deuterostomia	Protostomia	Placozoa	Porifera	Proteobacteria	Actinobacteria
	best Blast hit	XP_028408364.1 uncharacterized protein LOC114530941	IABP01004777.1 TSA: contig: c37863_g1_i1	XP_020615720.1 GPI inositol-deacylase-like	XP_027044245.1 acid-sensing ion channel 5-like	XP_012559638.1 PREDICTED: neurobeachin-like protein 2	XP_031605132.1 integrin alpha-X-like	XP_001901130.1 Phosphatase regulatory subunit family protein	XP_002110194.1 predicted protein	XP_019854518.1 PREDICTED: uncharacterized protein LOC109583549	WP_090491609.1 hypothetical protein	WP_014690906.1 MULTISPECIES: hypothetical protein
	organism	*Dendronephthya gigantea*	*Heliopora coerulea*	*Orbicella faveolata*	*Pocillopora damicornis*	*Hydra vulgaris*	*Oreochromis aureus*	*Brugia malayi*	*Trichoplax adhaerens*	*Amphimedon queenslandica*	*Myxococcus virescens*	*Actinoplanes sp.*
	"e-value"	1,00E-65		15	17	2,3	48	1,6	8,5	3,7	1,00E-28	7,00E-17

b

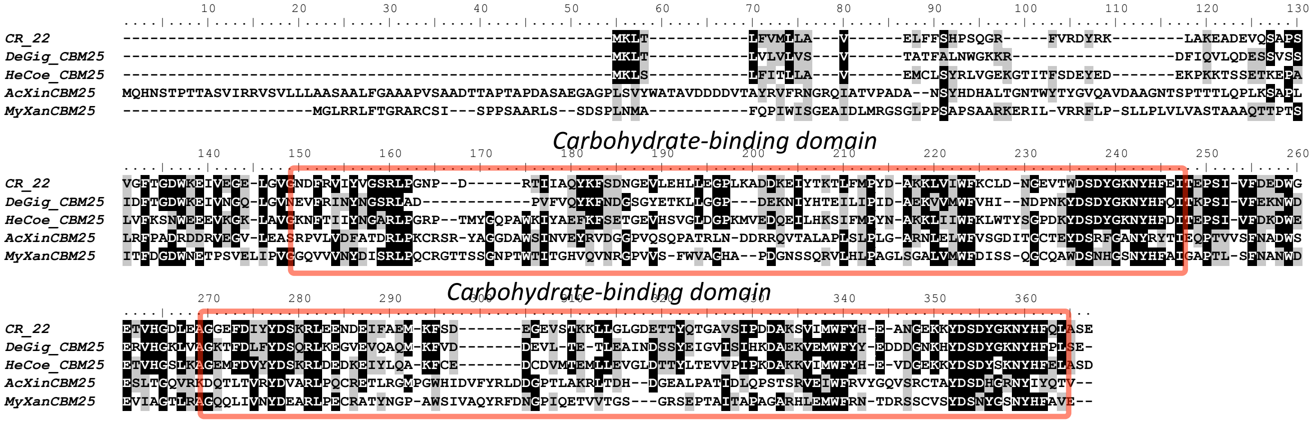

c

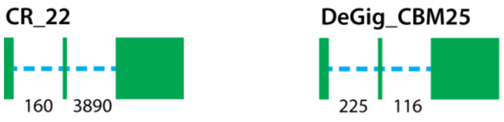

d

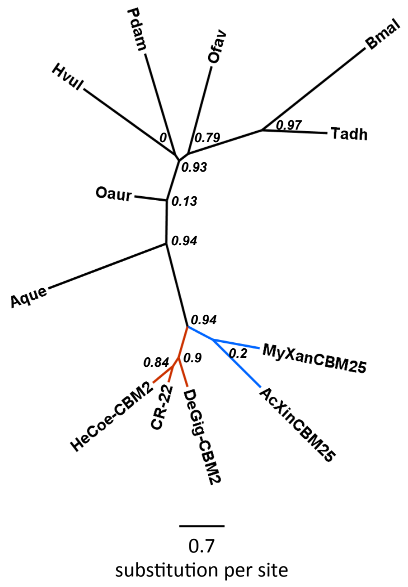

Supplement: Supplementary file 9 — Additional file 9: CR_22 as a gene horizontally transferred from unicellular organisms. a. Results of the BLAST search in the different taxa in NCBI database (Porifera, Placozoa, Cnidaria/Octocorallia, Cnidaria/Hexacorallia, Cnidaria/Hydrozoa, Protostomia, Deuterostomia and Bacteria). b. Multiple alignment of CR_22 with its 2 octocorallian homologs (HeCoe: Heliopora coerulea; DeGig: Dendronephthya gigantea) and 2 prokaryotes (MyXan: Myxococcus xanthus; AcXin: Actinoplanes xinjiangensis). The two carbohydrate-binding domains CBM25 (Pfam) of CR_22 are framed in red box. c. Genomic exon (green squares)/intron (dashed blue lines) structure of the CR_22 and DeGig_CBM25 gene coding sequences. Numbers correspond to the introns’ sizes in base pair. d. Phylogenetic tree (PhyML) of CR_22 homologs and proteins corresponding to first BLAST hit from a. [file 12862_2020_1734_MOESM9_ESM.pdf]
