# Supplementary material for: The skeletome of the red coral Corallium rubrum indicates an independent evolution of biomineralization process in octocorals
Source: BMC Ecol Evol. 2021 Jan 11;21:1. doi: 10.1186/s12862-020-01734-0 (PMC7853314; doi:10.1186/s12862-020-01734-0)
Supplement: Supplementary file 12 — Additional file 12: Galaxin-like proteins. The published protein sequences for galaxin homologs from Acropora millepora, Galaxea fascicularis, Stylophora pistillata and Euprymna scolopes [16, 48, 96–98], as well as the present Corallium rubrum CR_14 and CR_27 were submitted to the MEME discovery motif and the MAST motif scanning programs (http://meme-suite.org/). The motifs is a double di-cysteine motif similar to galaxin motif found by Reyes-Bermundez and colleagues [48], and is shown as logo on top. On the listed fasta sequences, this double di-cysteine motif, or galaxin motif, is highlighted in yellow in each sequence. The signal peptide sequence (http://www.cbs.dtu.dk/services/SignalP-5.0/) is in italic and underlined. [file 12862_2020_1734_MOESM12_ESM.pdf]

Additional file 12

MEME discovered motif: 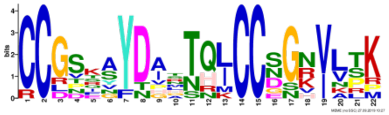

>CrubCR_14
*MKRLFICVCFFVLYTANP*AVVLNTEAKSTIHRSVFGVRKTRSVGAGAMYCCDDTMYNSSIHICCDDKLLSRRPGQNQVCCDDKSFDAMNNICCDGKILSRGNGKNSCCDDRNYNANTHVCCKDKILATAGGKTSCCDDKNYNPTF
DICCDKKILNLGSGPSSCCGDRLFNPSSKVCCDGKIRNVVGGKTSCCGDKTYNPTFNICCDKKIQSLGTGLRSCCGDKTYNPTTHVCCDDDISRIGAGKTACCDEDNYNPNTHVCCDDDILRIGADKKTACCDEDNYNPTTHVCC
DEHIMIIGAGKTACCDDKNYNPTTHVCCDDKIRSV

>CrubCR_27
*MNYLLVYAVWFVVFFTANA*IVIKPEPIPDPERRSCCGDRNFYLPNLFICCEDKILARHGSLNSCCGDNNYDSKINICCEDEIWRRRHGPNACCGPEVYNPKYYVCCDGEILSIENGRNSCCGAMNYNAKTHVCCDEEIRPIGAGA
TSCCVQDNYNSNTHVCCDEKILRIVGGRTTCCNDVAYNALTHVCCGGSILRKNGVHTACCDDKNFNPQYHVCCVDNLLSTATGRTACCGSTNYDNTTHVCCDHTIINLYEHWGGNSCCLNKNYDATTHLCCGGKVLSMLGGQTLC
CGDMNYSAKTHVCCRDKILSIKNHGNNTSCCGVEIYDPKSHVCCDGNILEV

>GfasGal_BAC41519 [Galaxea fascicularis]
*MSPTVSICFCSALFAVFSSCAS*FPRDTLSADSENVPNKLETRYRRQAPVPPVVSYCGGAPFSTATHICCNGNAEPKTGSTPMCCDSNSYDPLSQICCEGTVSHKATSPGAMPACCASDGYDMSTQLCCNDNVMHKPTGPTALPGC
CGDHSYDASVQLCCDSNVVPKMGSLSACCGPNSYDTNTTLCCDSNVAFVSGPQAQCCGSQGYDGATQLCCDSNVLPKPGATGACCGSQSYTQDTHLCCEGVIVLKAGPSFACCGSASYNQSSSLCCGATVVAKTPSKPVCCGSTS
YNPVTEICCDGHVGTRAGLTSPTCCGGAVFDAATAKCCDGVPVFNVPSCAGLA

>AmilGal1_D9IQ16
*MKPSGAFLSLCVVLLSLATHCFS*FPSDSLRRDAHSDTNALKSRDRRQAPAPQLSCGGVLYNPAAEMCCHGNVEPRVGASPMCCESSSYDPSTQMCCEGTVSNKPPGIAMCCGSEAYDANSQICCNGNINTKATGPTAQPGCCGEF
SYDAASQLCCDSHPVLMVGSLPSCCGRNGYDANTSLCCGDNNVAFVSGPQAACCGDMGYNRNTHLCCDSNVLPMPAMGACCGSWTYSQQTHLCCEGVQLYKGMNTGCCGAVGYNQVNSLCCEGTVVPKSPSKPVCCGTTSYNPLT
ELCCDGIAFFKTGFIRPTCCGGAIYDATVARCCDGVPTYNVASCAGLA

>AmilGal2_B8UU51
*MTRFTSIGLCAVLLFNVCSC*ATLQKDTIASMLKKGNSPRVTRQRRQLPSPCGSLQPGQLCCDSYKYNPVTHLCCNDNPAVKPASPTAIPGCCDQSAYDRNTHLCCDATLSPHPPATTLPACCGPVVYDSSVNSTQLCCAGAVLNK
PVGVPRALCCGTATYNPATQVCCMGFPVPKAGGPNATSLCCGPFSYDISTQMCCNGNIALKSATHTHCCGMFSFNPATHLCCNGYPYPKLGFISPSCCGSLVYDTLTMRCCDGSHVVLITPNQDPCANLA

>AmilGalLike1_ADI50284.1 galaxin-like 1 [Acropora millepora]
*MGLRRSIFILVAVFAVLQAAWA*GDKAYSIEDSLDKYDNAQAKLEDLDNTTAEDNIKEETSLLEETQDQDNEDQTDEQNDAEDEKISHDDDEEDADDEETSEAEDDAENEETADYSVDIPDDEETSDAEDGDADDFEDVGDSEDYL
DKKETTDADDYSDDEEATDDEAFPEDEETIEVEDFPEDNKIDFDGVDVADEINADPSQHGNEDSSDMTASKRTLVALCGRIRYIPSKQRCCNRRVIPRHLPCKPKCRTKYYNPYSHKCCFGRIVTPKPRPCLLGCGRRYYNPLTH
KCCFGRVVTPRSRPCRSVCGSKSYNPLTHRCCFGRVVTSKLRLCPLRCGVRYYNPLTHKCCFGRVVTPRSRPCSRLCGSKYYNPLTHKCCFGRVVTPKLRPCLLRCGVRYYNPLTHKCCSGRVVTPRSSPCRTVCGLKYYNPLTH
RCCFGRVVTPKSRPCPLRCGVRYYNPLTHKCCFGRVVTPRLRPCLLRCGVRYYNPLTHKCCFGRVVTPRLRPCLLRCGVRYYNPLTHKCCLGRVVTPKLRPCLLRCGVTSYNPITQKCCSRRLVISKLRPCPLKCGSRYYYPTNQ
KCCFGHVVKPKTSPCLLRCGLKFYNPLTFKCCVGNIVTPKPFSCPLPCGATYYKPVSHRCCYGTVILKSFKCVIPPYKPR

>AmilGalLike2_ADI50285.1 galaxin-like 2 [Acropora millepora]
*MMSMVRGIFVGLLVLAFAATLRA*ETFEEDSQSNPEQAADTNEQVKAADASAQELAADPSERKIEVDSNEPETEADPSEQETEADSSEQETEADPSQQETEADPSAEQGAADPGEQKAEVDLDEQETKADPNEQETESDPNEEETE
ADPSEHETEADSSEREREADASEQEAADISEQETEVDTNEQETEADQSEQETAADPSEHETEADPSEQEQAADSSLQQEVADSRVEFAADLSFQEPFDTDQAFANDTSDYDESNKKKGDNFLEGSETEKRSVGYCNKISYSKTTQ
FCCLDRIHPKTPLGRPGLCCGSSVYTIGTQLCCSGRVLTKNSTINACCGTQGYNIRTHRCCGRTLYNRNTQLCCQGRILPKRSTINACCGTQGYNIRTHRCCRRTLYNRNTQLCCQGRIISKNSTINACCGTQGYNIRTHRCCGR
TLYNRNTQLCCQGRIISKSSTINACCGTQGYNISTHSCCRRTLYNRNTQLCCYGRIHAKTLSRKTRLCCGSSSYTTTTHLCCGGRVYNRNSYSLCCGTGLYNRRVQGCCRGRSVYTLKRQKCCTKGVIPSWASCYCDNGHGPYIS
IP

>SpisGal_iso06123 [Stylophora pistillata]
*MWYVIATCLCTVLLVTLTEG*ASFKKDHALQERNEQRMGPNKGVSRFRRQMAICGNQYYYPGFALCCDDNIVPIIPGQASMCCGSSVFSPLIQICCDANPHSKIGLQRPACCGSDPYDANNTLCCDTTPLPMVGVQSACCGSDIYN
MATQICCDSYVVMKPGINSVCCGSQAYDIDTQLCCEGRPVPKVGNFSACCGSTAYDVHNQLCCENHVVPKVSPYSACCASQGQVIFSYNTLTQLCCEGFIYPRAPGVQCCGSLMYNTMTYMCCDGSLILPKLITGCAGFA

>SpisGalLike_iso11429 [Stylophora pistillata]
MWGGELQSLDPQVLLQQSGSKSFICPAKCGYRNYNPWTQKCCYGRIVSKRFSCRFRCGFRSYNPRTHKCCFNRLVVRSVTCPVKAFAALSATIHGSKSVAMAK

>Esco_AIT11913.1 galaxin 1 [Euprymna scolopes]
*MMKIATLLCVMFGLLA*RRIRRRDCGNRTYDPQFQICCSGKVRTRIGSYTKCCRTIAYDHRTRICCSGVVKRKPGINTGRCCRYNVYNSQYALCCAGRVTMKPTKRSACCKYRAYDTDNFRCFKGKILPK
